# Estimating Ancestral States of Complex Characters: A Case Study on the Evolution of Feathers

**DOI:** 10.1093/sysbio/syaf063

**Published:** 2025-09-13

**Authors:** Pierre Cockx, Michael J Benton, Joseph N Keating

**Affiliations:** Key Laboratory of Vertebrate Evolution and Human Origins, Institute of Vertebrate Paleontology and Paleoanthropology, Chinese Academy of Sciences 142 Xizhimenwai Str, Beijing 100044, China; School of Earth Sciences, Life Sciences Building, University of Bristol, Tyndall Avenue, Bristol BS8 1TQ, UK; School of Earth Sciences, Life Sciences Building, University of Bristol, Tyndall Avenue, Bristol BS8 1TQ, UK; School of Earth Sciences, Life Sciences Building, University of Bristol, Tyndall Avenue, Bristol BS8 1TQ, UK

**Keywords:** Ancestral state estimation, complex character, cross-validation, dinosaurs, feathers, maximum likelihood, phylogenetic comparative methods

## Abstract

Feathers are a key novelty underpinning the evolutionary success of birds, yet the origin of feathers remains poorly understood. Debates about feather evolution hinge upon whether filamentous integument has evolved once or multiple times independently on the lineage, leading to modern birds. These contradictory results stem from methodological differences in statistical ancestral state estimates. Here, we conduct a comprehensive comparison of ancestral state estimation (ASE) methodologies applied to stem-group birds, testing the role of outgroup inclusion, tree time-scaling method, model choice, and character coding strategy. Models are compared based on their Akaike Information Criteria (AIC), mutual information, as well as the uncertainty of marginal ancestral state estimates. Our results demonstrate that ancestral state estimates of stem-bird integument are strongly influenced by tree time-scaling method, outgroup selection, and model choice, whereas character coding strategy seems to have less effect on the ancestral estimates produced. We identify the best fitting and most generalizable models using AIC scores and leave-one-out cross-validation (LOOCV), respectively. Our analyses broadly support the independent origin of filamentous integument in dinosaurs and pterosaurs and support a younger evolutionary origin of feathers than has been suggested previously. In terms of model selection, we observe little correlation between AIC or corrected AIC (AICc) and LOOCV error, suggesting that, for our data set, model fit does not reliably predict generalizability. However, both approaches favor models that infer a similar pattern of feather evolution. More globally, our study highlights that special care must be taken in selecting the outgroup, tree, and model when conducting ASE analyses.

Feathers are a key novelty underpinning the evolution of avian flight. As such, knowledge of the sequence and timing of feather evolution is essential for understanding the origin of the avian body plan. Exceptionally preserved stem-avians from the Late Jurassic–Early Cretaceous provide unparalleled insight into the evolutionary origin of feathers. Filamentous integuments with diverse morphologies have been documented in numerous dinosaur taxa (e.g., O’Connor 2020; Xu 2020) and even in pterosaurs (e.g., Kellner et al. 2010; Yang et al. 2019; Cincotta et al. 2022). These morphotypes range from simple structures to more complex ones and are associated with specific body regions and functions. Crucially, they are non-randomly distributed across the avian stem; the simplest morphotypes are widespread whereas more complex ones (such as pennaceous feathers) are restricted to taxa more closely related to the crown group (Benton et al. 2019).

To date, at least 13 distinct morphotypes of feathers have been recognized among integumentary structures reported from non-avian dinosaurs. Two basic categories of feathers are commonly recognized based on the general feather structure: pennaceous and plumulaceous types. Pennaceous feathers are characterized by barbs fused to a central shaft called the “rachis,” thus defining a clear planar surface (the vane). Plumulaceous feathers have no vane and are characterized by a rudimentary rachis and a tuft of barbs. Beyond this simple distinction, feather morphotypes can be further categorized according to various criteria including number of filaments (single or multiple), general morphology, secondary branching (e.g., barbules allowing interlocking of the barbs through hooklets), and symmetry of the vanes. This leads to the following categories: monofilaments, basally joined filamentous feathers, filamentous feathers with a central filament, pennaceous feathers, and asymmetrical pennaceous feathers. The first three categories mentioned include the filamentous morphotypes of feathers.

Testing hypotheses about the timing, sequence, and rate of integument evolution in early avians requires rationalization of the distribution of feather morphotypes within a phylogenetic framework (Fig. 1). To this end, statistical likelihood-based ancestral state estimation (ASE) methods have been applied. These methods utilize three components: a phylogenetic tree with branch lengths representing time or evolutionary rate; an evolutionary model describing the process by which a trait changes through time; and a set of observed character states corresponding to the tree tips (i.e., observed states in extant or fossil taxa). For discrete characters, this is typically a Markov model describing the transition rates between the character states. Unfortunately, ASE studies applied to early avian integument have yielded contradictory results, supporting either a single early origin of feathers around 250–240 Ma (Yang et al. 2019; Cincotta et al. 2022) or else multiple origins of filamentous integument around 160 Ma (Campione et al. 2020). Contradictory results stem from methodological differences between studies relating to one of the three components of ASE.

**Figure 1.**
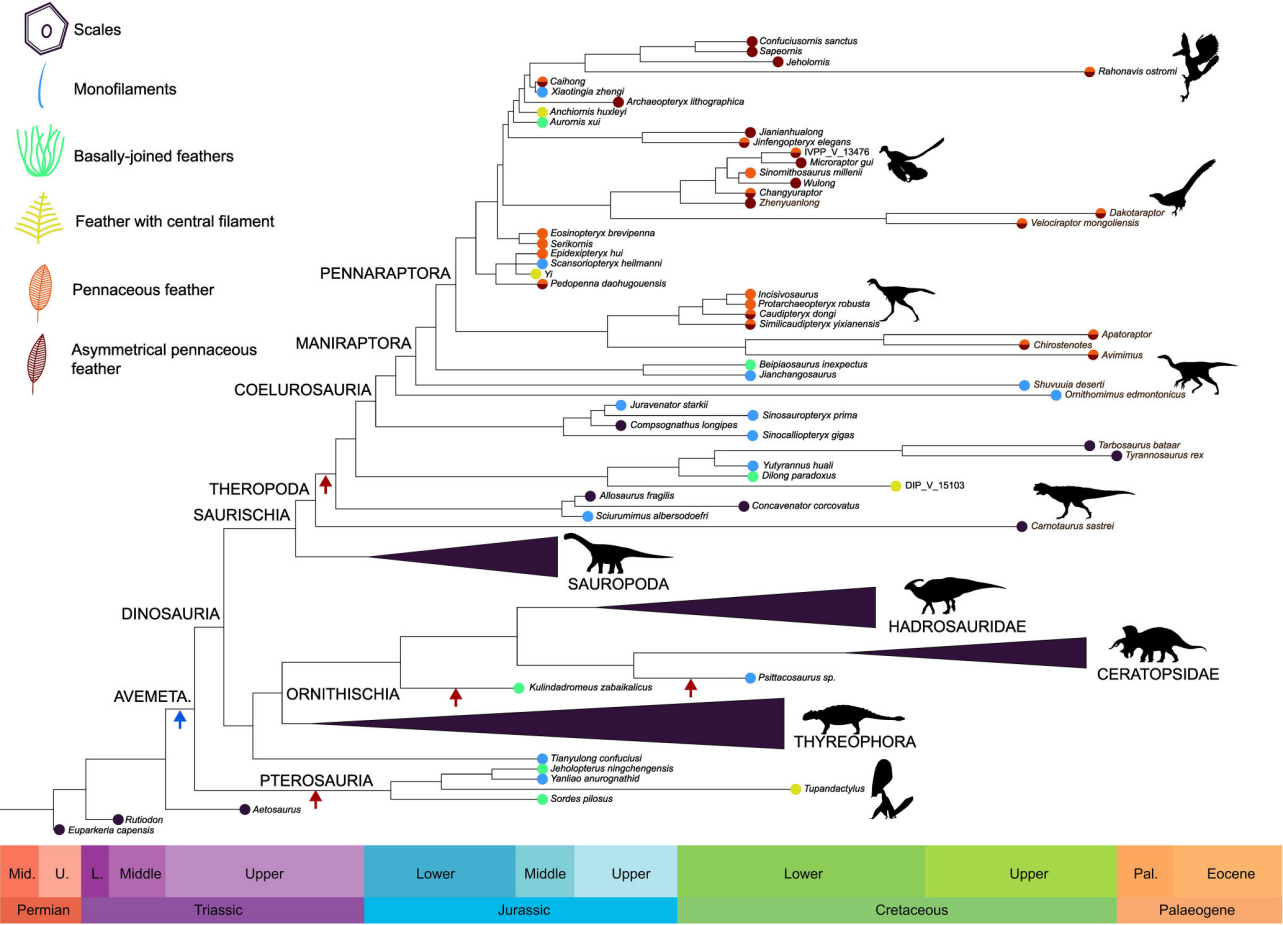
Feather evolution. The phylogenetic tree is plotted against geological time with silhouettes illustrating the main groups of dinosaurs as well as Pterosauria. Drawings illustrate the feather morphotypes representative of the character states used in this study. Currently, there are two major competing hypotheses for the timing of feather origin: an early hypothesis (blue arrow) in the Triassic implying a single origin of feathers, and a late hypothesis (red arrows) in the Jurassic implying several independent origins (within Pterosauria and Dinosauria). Silhouettes from PhyloPic.org. *Camarasaurus supremus* by Matthew Wedel and *Caudipteryx zoui* by Scott Hartman are licensed under CC BY 3.0 (https://creativecommons.org/licenses/by/3.0/), whereas *Carnotaurus sastrei* by Scott Hartman is licensed under CC BY-NC-SA 3.0 (https://creativecommons.org/licenses/by-nc-sa/3.0/); all others are in the public domain (CC0 or PDM 1.0).

The first component is the phylogenetic tree. When selecting a tree, topology, choice of outgroup(s), and branch lengths are crucial factors to consider. New fossils may change our understanding of relationships on the bird stem-lineage. Uncertainty in the topology leads to competing tree hypotheses characterized by different distributions of the tip states. Consequently, ASE results are impacted. Simulations from Duchêne and Lanfear (2015) showed that phylogenetic uncertainty results in a bias of the estimates of the number of evolutionary transitions between states. These results suggest that the number of transitions would be likely to be overestimated in empirical studies. Mooers and Schluter (1999) identified two major problems arising from outgroup selection. First, each branch of a phylogenetic tree provides information about the rates of change. When entire clades are represented by a single lineage, as it is often the case for an outgroup, the estimates of rate may be significantly biased. Second, assuming that transition rates are homogenous between the ingroup and the outgroup is unrealistic, particularly if the outgroup is phylogenetically distant from the ingroup. Multiple outgroups can contribute to a more robust estimation of the ancestral condition of the ingroup (Wright et al. 2015). The choice of method for estimating branch length can also have a significant effect on ASE results. Previous studies, based on simulations and empirical data, have identified that ASE is sensitive to branch lengths (Litsios and Salamin 2012; Cusimano and Renner 2014). Wilson et al. (2022) further confirmed these findings and suggested using model-fit statistics (such as corrected Akaike Information Criteria (AICc) or Bayesian Information Criterion (BIC)) to compare trees with alternate branch lengths set and evolutionary models. The combination associated with the best model fit should then be selected. In ASE studies incorporating fossils, branch lengths are commonly estimated using a posteriori time-scaling methods such as the “equal” method or the “minimum branch length” (mbl) method (Laurin 2004; Brusatte et al. 2008). These methods avoid zero-length branches either by taking an equal share of preceding non-zero length branches (equal method) or by setting an mbl method.

The second component is the evolutionary model. There is a plethora of evolutionary models to select from, and choosing the most appropriate is a significant challenge. The simplest model, the Equal Rates (ER) model, assumes that all state transitions occur at the same rate. More complex parameterized models, such as the Symmetric (SYM) and All-Rates-Different (ARD) models, relax this assumption, allowing transition rates to vary. More recently, Hidden Markov models (HMM) have been applied to ASE. These models link hidden states to observed states via many-to-one mapping (i.e., multiple hidden states lead to the same observed state), permitting modeling of more complex evolutionary processes. Under the Hidden Rates Model (HRM) (Beaulieu et al. 2013; Boyko and Beaulieu 2021), observed data can result from several processes occurring in different parts of the phylogeny, linked via the parameter process (Boyko and Beaulieu 2021). Tarasov (2019) proposed using Structured Markov Models (SMM) in addressing the problem of modeling hierarchical character-state dependencies, such as the classic tail color problem highlighted by Maddison (1993). Through incorporation of hidden states in an SMM, it is possible to account for both the hierarchical processes resulting from character dependencies, together with hidden processes, such as gene regulatory evolution (Tarasov 2019). Alternatively, hierarchical character dependencies can be embedded within the model without requiring hidden states via appropriate amalgamation of the rate matrices (Tarasov 2023).

The third and final component is the trait data. For discrete ASE, this comprises categorical character-state observations for each tip of the tree. However, defining morphological characters and states is rarely straightforward and often subjective. Different character states can manifest in the same organism at different life stages and/or in different body regions. This is particularly true when character states are developmentally related, as is the case with feather morphotypes (e.g., Lin et al. 2020). Character states are also frequently defined with differing levels of granularity (e.g., Yang et al. 2019; Campione et al. 2020) and it is currently unclear whether it is preferable to use broad or specific character-state definitions. The effect of character coding scheme on statistical ASE is poorly understood. Applying alternate coding schemes results in equivalent characters with differing numbers of states and/or different state definitions. Alternate coding schemes will therefore likely require different evolutionary models and potentially yield different evolutionary inferences.

In this study, we conduct a series of ASE analyses to evaluate the effect of outgroup selection, time-scaling method, model selection, and character coding scheme on ancestral state estimates of early avian integumentary structures. We focus on the origin(s) of feathers and the transitions between major categories of feathers defined based on their general morphologies. We evaluate the relative performance of different approaches using model fitness and a leave-one-out cross-validation approach to find the most generalizable models. Through these comprehensive analyses, we identify a robust set of ancestral state estimates clarifying the timing and sequence of early feather evolution. More generally, our research provides an important case study for best practices in ASE analyses of complex traits, namely traits with ambiguous character states, hierarchical dependent relationships between states and/or nonlinear paths of possible state transitions.

## Materials and Methods

### Data Collection and Character Coding

To conduct a phylogenetic ASE study, we collected data on plumage characters across dinosaurs and pterosaurs from a total of 94 taxa. Character states were determined through a literature review (see Supplementary Table S6). When more than one morphotype of feathers was reported from a taxon, only the most developmentally complex was considered. Previous analyses have shown that coding multiple trait values for taxa preserving several feather morphotypes did not allow the recovery of much information on patterns of evolution of feather types (Yang et al. 2019). This is probably because this approach does not necessarily consider similar morphotypes found in different taxa as homologous. For instance, taxa coded as having pennaceous feathers and other morphotypes were treated separately: the different combinations of feather morphotypes led to independent states. The nomenclature used for distinguishing feather types based on their general morphology follows Xu (2020). Analyses were performed using a revised version of the phylogenetic tree of Yang et al. (2019) and incorporating additional taxa by editing the tree in Mesquite (Maddison and Maddison 2023).

### Phylogenetic Time Calibration

Basic data on first appearance datum (FAD) and last appearance datum (LAD) for each taxon were taken from Yang et al. (2019). We collected FAD and LAD of the taxa added to the tree using numerical ages for the boundaries of the stratigraphic units in which they were documented. Whenever possible, we used absolute dates reported in the scientific literature, and the International Chronostratigraphic Chart 2023-06 (Cohen et al. 2023) served as a reference otherwise. Branch lengths were then estimated using the timePaleoPhy function of the paleotree R package (Bapst 2012) and the DatePhylo function of the strap package (Bell and Lloyd 2015). The equal branch length (equal) and mbl methods were used. These methods (DatePhylo and timePaleoPhy) lead to distinct dated trees (i.e., same topology but different branch lengths) owing to a different ordering algorithm used. In both cases, we set the root length to 1 myr. We used the “equal_paleotree_legacy” argument in the case of timePaleoPhy. Phylogenetic trees with pie charts displaying the ancestral likelihoods were produced using the nodelabels() and tiplabels() functions from the ape R package (Paradis and Schliep 2019).

### Coding Schemes and Models

Ancestral states were estimated using marginal likelihood estimation. We used the corHMM function of the package of the same name (Boyko and Beaulieu 2021). First, we applied simple Markov models to each coding scheme, treating character states as independent. State transitions were modeled using the ER, SYM, and ARD transition rate models.

Plumage data were coded following three distinct schemes with increasing numbers of character states (Table 1). Coding 1 is a simple binary character with the states “Scales” and “Feathers” (Coding 1). Coding 2 relies on a three-state character introducing a distinction between filamentous and pennaceous feather morphotypes. Finally, Coding 3 is a six-state character allowing to further distinguish the main categories of feather morphology. The character matrix contains multiple characters coded as uncertain in some taxa. In such instances, equal probability of the possible states was assigned.

**Table 1.** Feather coding strategies and models tested in this study

Coding strategies	Character states	Coding schemes	State space
Coding 1	Scales; feathers	All models: {0,1}	{0,1}
Coding 2	Scales; filamentous feathers; pennaceous feathers	Mk: {0,1,2} ED: {0,1}; {0,1} SMM: {0,1}; {0,1} HRM: {0,1,2}	{0,1,2} {0,1,2} {0&1,10,11} {00,01,10,11}
Coding 3	Scales; monofilamentous integument; basally joined feather; feather with central filament; pennaceous feather; asymmetrical pennaceous feather.	Mk: {0,1,2,3,4,5} ED: {0,1}; {0,1}, {0,1}, {0,1}, {0,1} SMM: {0,1}, {0,1}, {0,1}, {0,1}, {0,1} HRM: {0,1,2,3,4,5}	{0,1,2,3,4,5} {0,1,2,3,4,5} {0&1&1&1&1&1,10,000. . .} {00,000,00,001,00,010. . .}

Note: Mk = standard Markov models; ED = embedded dependency; SMM = Structured Markov Model; HRM = Hidden Rates Model. Three coding strategies with varying states definitions were tested. These involve different coding schemes and corresponding state space depending on the model used.

We then applied two methods developed for modeling dependent characters: embedded dependency (ED) and SMMs. These two approaches require different coding schemes, and result in different state spaces. The ED model involves the amalgamation of the rate matrices of the characters (Tarasov 2023). In other words, this involves merging two or more individual characters with a dependent relationship into a single character. For instance, in the case of Coding 2, the coding scheme involves a controlling character (feathers: 0, absent; 1, present) and a dependent one (feather structure: 0, plumulaceous; 1, pennaceous). Amalgamation of the rate matrices results in a single character with three states: absences of feathers (0), plumulaceous feathers (1), and pennaceous feathers (2). Two variants of the ED model can be used according to the type of character involved in the hierarchy: qualitative (ED-QL) and birth–death (ED-BD). They differ in the way the rate matrices are parameterized and whether certain transitions are permitted. The ED-QL process models the evolution of a dependent character and its inherent qualitative property. For example, using the “tail color” example of Maddison (1993), a tail may be absent or present. If the tail is present, it may be red or blue. Because tail color is an inherent property of the tail, it logically follows that when a tail evolves, it already has a color, either red or blue. This is the simplest type of ED, but another situation may arise. To illustrate the ED-BD process, Tarasov (2023) proposed the hypothetical case of tail armor evolution in species without tails, with unarmored tails and with armored tails. In this example, evolving armored tails from an ancestral state of tail absence implies two trait “births”: the evolution of a tail and the evolution of tail armor. Under the ED-BD model, transitions between states that would imply two or more trait “births” are forbidden. As such ED-BD model assumes lags between the evolution of anatomically dependent structures.

SMM and HMM are derived from traditional Markov models. SMMs equipped with hidden states assume that the evolution of the observed dependent character states is a result of an unobserved evolutionary process such as gene regulatory evolution (Tarasov 2019, 2020). Such SMMs are formed by amalgamating the characters as independently evolving (SMM-ind) or by imposing stricter relationships reflecting the hierarchy of the traits (SMM-switch). The approach is similar to that of the ED models, but absence states are represented by two or more hidden states. Thus, in the example of Coding 2, feather absence (scales) is represented by states {00} and {01}. These hidden states can be interpreted as feather absence with a liability toward plumulaceous or pennaceous feathers, respectively. The ED and SMM transition rate matrices were built using the rphenoscate package with the amaED and amaSMM functions, respectively (Porto et al. 2023). SMMs were run with the argument “collapse” of corHMM set to FALSE. This setting ensures that the unobserved state combinations are modeled. Finally, we applied the hidden rates model (Boyko and Beaulieu 2021) to Coding scheme 3. This model accounts for heterogeneity of the transition rates among lineages (Beaulieu et al. 2013). We used two rate categories (allowing modeling of “slow” and “fast” transition rates) and three rate categories (allowing modeling of “slow,” “intermediate,” and “fast” rates).

**Figure 2.**
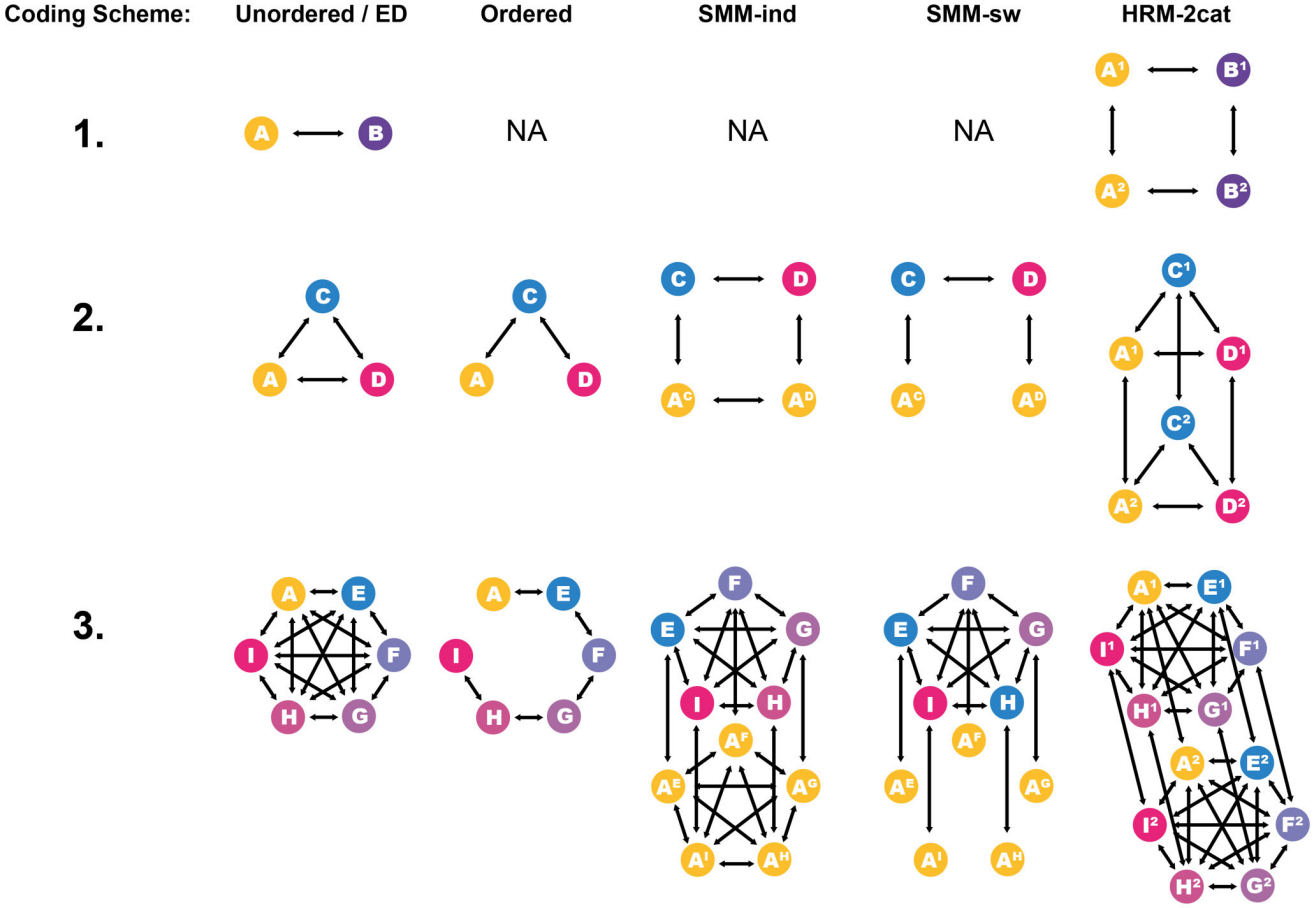
Diagrammatic representations of the different evolutionary models and coding schemes. Each letter refers to a state whereas the arrows show the possible transitions. A = scales; B = feather; C = filamentous feather; D = pennaceous feather; E = monofilamentous integument; F = basally joined feather; G = feather with central filament; H = pennaceous feather; I = asymmetrical pennaceous feather. Superscript letters are used in the hidden layer of the SMM to denote liabilities. Superscript numbers in the HRM indicate the rate categories (either 1 = slow or 2 = fast). For clarity, the HRM-3cat model is not shown in this figure.

Table 1 summarizes the different coding strategies and the associated coding schemes and state spaces. Diagrammatic representations of the different evolutionary models and coding schemes illustrate the states and possible transitions (Fig. 2), as well as the associated rate parameters (Supplementary Fig. S1).

### Assessing the Sensitivity of Maximum Likelihood Analyses to a Series of Parameters

#### Experiment 1

Outgroups are highly influential in ASE. Their character states can strongly affect ancestral estimates, particularly at the root due to their phylogenetic proximity. In addition to the choice of outgroup, factors such as the number of outgroups, their phylogenetic position relative to the ingroup, and whether they are extinct (e.g., fossil taxa) are also important considerations. Choice of outgroup is especially important when dealing with time trees, as is the case with all our analyses. In such cases, fossil outgroups will (by definition) sit upon shorter branch lengths and exert more influence on the ASE. The impact of outgroup selection was evaluated by running successive analyses with outgroups formed of 1 fossil taxon (Outgroup 1), 2 fossil taxa (Outgroup 2), 3 fossil taxa (Outgroup 3), and 2 fossil taxa plus a modern taxon (Outgroup 4) (Table 2). Coding strategy 3 (see Table 1) was used and the analyses were run with an equal tree produced with the timePaleoPhy function under unordered models. As for Experiments 2 and 3 described below, the ER results do not differ strongly from the SYM results. For Experiments 1–3, we focus on SYM and ARD results in the main text, whereas the ER results are presented in the Supplementary Material (SupplementaryTables S1–S3 and Supplementary Figs. S2–S4).

**Table 2.** Outgroups evaluated in this study

Outgroup evaluated	Taxa
Outgroup 1	*Euparkeria*
Outgroup 2	*Euparkeria, Rutiodon*
Outgroup 3	*Aetosaurus, Rutiodon, Euparkeria*
Outgroup 4	*Euparkeria, Rutiodon, Crocodylus*

Note: *Crocodylus* represents the only extant lineage.

#### Experiment 2

We tested the effect of a posteriori time-scaling methods by running the analyses with different time-scaled trees. We used the equal (DatePhylo); equal (timePaleoPhy); and mbl methods. To ensure the results are comparable, the analyses were performed with the same character coding strategy (Coding 3) and outgroup (Outgroup 3), using the same models (unordered models). Coding 3 was selected as it includes multiple feather categories, thus providing additional details about the sequence of evolution of the main feather morphologies. We selected Outgroup 3 as this is the case where the model and tips assign the highest amount of information to the avemetatarsalian node (see the Results section), which is the most recent common ancestor of pterosaurs and dinosaurs. Identifying its ancestral state is of major concern to feather evolution studies, as this can shed some light on a possible single evolutionary origin of feathers.

#### Experiment 3

We also assessed the impact of the coding strategy on our results by testing three coding strategies ranging from a simple binary character to a more complex six-state trait, as described earlier (Table 1). Analyses were run with Outgroup 3 and an equal timePaleoPhy tree. Such tree was selected as it is associated with the lowest AICc under each of the transition rate model tested (see the Results section).

#### Experiment 4

We ran the analyses under 21 different evolutionary models, incorporating various assumptions about transition rates, rate heterogeneity, and hierarchical dependent structure. As in Experiment 2, all the analyses were run with Coding strategy 3 and Outgroup 3. Initially, we used an equal (timePaleoPhy) tree (see the Experiment 4: Effect of the Model section). We then repeated the analysis using an equal (DatePhylo) and mbl tree to ensure that our results were robust to different time-scaling methods (see the Tree/Model Combinations section).

### Measuring Model Fit, Uncertainty, and Information

In all experiments, models were compared in terms of their AIC and AICc, their uncertainty, and, when appropriate, their mutual information. In the case of AICc, we have decided to exclude the ARD HRM (3) models from consideration due to this model being overparameterized (the number of free parameters is equal to the number of tips). This leads to very low (negative) values that are not interpretable. We used the ggplot2 R package to produce a plot comparing the models based on these statistics (Wickham 2016).

Raw uncertainty (Keating 2023) is based on the raw error measure, established by Holland et al. (2020). The calculation involves summing the highest ancestral state marginal estimates for each node, dividing the result by the total number of nodes, and finally, subtracting this value from 1. A theoretical absolute certainty in the ASE would always result in a value of 0. On the other hand, the lowest possible certainty (with an equal probability of each state at each node) would be a function of the number of states: as the number of states increases, the maximal uncertainty would tend to 1. To allow comparisons between models with different state spaces, the proportion of the maximal theoretical uncertainty can be calculated. Mutual information, a concept from information theory, can be applied to Markov models (Cover and Thomas 1991; Sober and Steel 2011).

As stated by Boyko and Beaulieu (2021), mutual information is the difference between the unconditional entropy of the node states and the entropy of the node states conditioned on the data. The unconditional entropy increases as the number of states in the data set increases and defines the upper limit of what can be learned. It is set by the model and is always the same for each node of the tree. The conditional entropy is calculated using the conditional probabilities that the nodes are fixed in the various states, given the probabilities of observing the tip data. In other words, mutual information is a metric representing the reduction in uncertainty about ancestral states. The corHMM function can return a vector comprising the amount of information the tip states and model assign to each node. We simply summed all the values to get the total information for the whole tree.

A cross-validation approach is a more exhaustive method than AIC for computing the fitness of a model (Duchêne et al. 2016). This method involves training the model on a subset of the data and then assessing model fit with another non-overlapping subset of the observations (e.g., Lartillot 2023). Several variants of such methods exist, but we used a leave-one-out cross-validation (LOOCV) approach where each observation is removed from the data set whereas the model is trained on the remaining data.

Because time-scaling methods may influence ancestral state estimates through changes in the branch lengths, we opted to assess generalizability across tree/model combinations. To balance sensitivity to actual generalizability and differences in robustness to variations introduced by different branch lengths, we defined a 10% threshold on LOOCV mean error. Combinations within 10% of the lowest mean error are treated as having a comparable generalizability according to fit to models. This approach emphasizes robustness across alternative phylogenetic trees while avoiding over-interpreting marginal differences in error.

For each of the 63 model–tree combinations, we performed the following actions: (1) Drop a tip; (2) conduct an ASE analysis; (3) save the estimated transition rates for the Markov model; (4) reincorporate the tip and set its state to unknown; (5) use the model with the transition rates previously estimated to estimate the state of the tip; and (6) calculate and save the error. The process is repeated for each of the tips of the trees and thus results in more than 6000 analyses in total. To conduct the LOOCV analyses, we used functions from the phytools R package (Revell 2012) because tip priors that can be used as indices for comparing the estimated states to the true states can be provided. The fitMk() function was used to fit the model, whereas ancestral states were estimated using the ancr() function. The argument “tips = TRUE” ensures estimated tip states are obtained, including unknowns. The LOOCV error of the model can be calculated for each tip by subtracting the estimated likelihoods for the right tip state from 1. This is equivalent to summing the likelihoods for all the incorrect tip states. A LOOCV mean error can then be calculated for the whole tree. The LOOCV method is implemented using the loo_cv() function from the *treesurgeon* package, available on the GitHub repository of one of the authors (J.N.K.; https://github.com/evo-palaeo/treesurgeon). It directly outputs the raw mean error and mean log-likelihood of the model.

Model selection using information criteria (AIC or AICc) requires the data to remain constant. Markov models do not involve joint estimation of trait evolution and topology/branch lengths: the phylogeny and branch lengths are part of the data. However, as Wilson et al. (2022) pointed out, branch length transformations are often conducted when fitting phylogenetic comparative models. In such cases, the branch lengths become part of the model because the transformation represents macroevolutionary dynamics and not branching times of the phylogeny. Ideally, the log-likelihood of the phylogeny should be added to the computation of the AIC when branch length differences originate from alternative phylogenies. Nonetheless, in the absence of any other evidence, AIC is appropriate to test alternative branch lengths such as in our Experiment 2 (Wilson et al. 2022). Experiment 1 involves different outgroups and as such, changes the number of taxa and the topology of the tree. Consequently, statistics such as AIC or AICc cannot be used for tree/model selection in this case. A similar conclusion can be drawn in the case of Experiment 3 where different coding strategies are tested. Changes in the definition of the states imply changes in the data, thus rendering AIC inapplicable for model selection in this experiment. We propose below alternative methods for selecting outgroups/models of our analyses whenever standard information criterion methods (e.g., AIC, AICc) cannot be used.

In the case of Experiment 1, the resulting trees are characterized by the same ingroup but different outgroups. These can be compared through a procedure similar to LOOCV as follows. For each tree, we iteratively dropped an ingroup taxon, fitted a model and saved the estimated transition rates, reincorporated the taxon, and finally estimated the ancestral states (including the tip initially removed) using the transition rate matrix previously estimated. We then calculated the average error by simply summing the tip state error of each iteration, divided by the number of ingroup tips. The value derived from this experiment represents the accuracy of the model in estimating the tip states of the ingroup. It can be used for comparing trees with different outgroups.

### Model Averaging

To account for model uncertainty in estimating the ancestral states, model averaging was used. We first calculated Akaike weights (Akaike 1978). To do so, for each tree/model combination, we calculated the differences in AIC, with the best combination. This can be written as


\begin{eqnarray*}
{\Delta }_i(AIC) = AI{C}_i - \min \,\,AIC.
\end{eqnarray*}

The weights can then be calculated as


\begin{eqnarray*}
{w}_i(AIC) = \frac{{exp\left( { - 0.5\Delta (AIC)} \right)}}{{\sum\nolimits_{\kappa = 1}^K {exp\left( { - 0.5\Delta (AIC)} \right)} }}.
\end{eqnarray*}

The ancestral likelihoods of each tree/model combination are finally multiplied by the corresponding weighting and summed across the 63 combinations. This results in averaged ancestral likelihoods. We calculated the weightings and plotted the ancestral likelihoods for AIC (Supplementary Fig. S5).

In the case of the LOOCV mean error, the weights were calculated by subtracting the error of a model from 1 and dividing the result by the sum of the results for all the models.

## Results

### Experiment 1: Effect of the Outgroups

**Figure 3.**
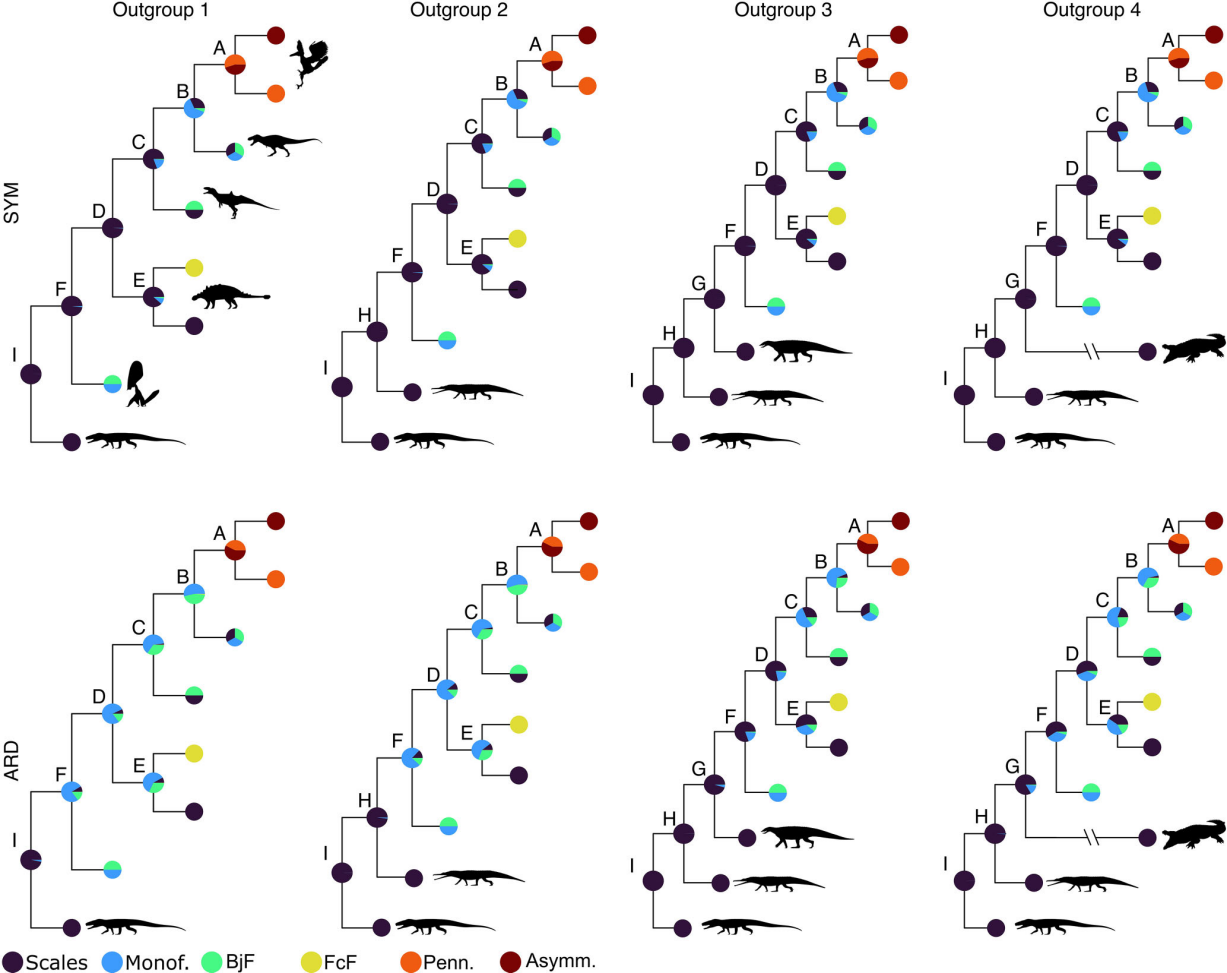
Comparison of the ancestral state likelihoods for four different outgroups selected and two transition rate models (SYM and ARD). A subset tree with only six nodes is plotted to facilitate the comparison. Node labels: Outgroup 1–A = most recent common ancestor (MRCA) of *Rahonavis* and *Sapeornis*; B = Coelurosauria; C = Tetanurae; D = Dinosauria; E = MRCA of *Kulindadromeus* and *Psittacosaurus*; F = Avemetatarsalia; G = MRCA of *Sordes pilosus* and *Aetosaurus* (Outgroup 3) or MRCA of *Sordes pilosus* and *Crocodylus* (Outgroup 4); H = MRCA of *Sordes pilosus* and *Rutiodon*; I = MRCA of *Sordes pilosus* and *Euparkeria capensis*. Abbreviations: Monof. = Monofilamentous integument; BjF = Basally joined filamentous feather; FcF = Feather with central filament; Penn. = Pennaceous feather; Asymm. = Asymmetrical pennaceous feather. Silhouettes from PhyloPic.org. *Euparkeria capensis* by Oliver Demuth is licensed under CC BY 4.0 (https://creativecommons.org/licenses/by/4.0/); *Paleorhinus* by Scott Hartman and *Euoplocephalus tutus* by Andrew A. Farke are licensed under CC BY 3.0 (https://creativecommons.org/licenses/by/3.0/). Other silhouettes are in the public domain (CC0 or PDM 1.0) or detailed in the Figure 1 caption.

The choice of outgroup does not appear to influence the ancestral state likelihoods in the case of the SYM model: the estimated ancestral likelihoods remain the same regardless of the outgroup tested (Fig. 3). The uncertainty of the results (Table 3) remains at about 0.15 for each tree with a different outgroup selected.

**Table 3.** Raw uncertainty calculated for each outgroup selected: with one fossil, two fossils, three fossils, and with two fossils and one extant taxon

	SYM	ARD
Outgroup 1	15.07	18.11
Outgroup 2	14.88	18.14
Outgroup 3	14.66	17.40
Outgroup 4	14.53	19.56

Note: Coding 3 is used here.

In the case of the ARD model, in contrast, outgroup choice does affect ancestral likelihoods. The result with a single outgroup was different from any of the analyses using the SYM model, noticeably placing more certainty in the presence of monofilaments early in feather evolution. Increasing the number of outgroups, either by addition of fossil taxa or by a mix of extant and fossil taxa, changed this result, bringing the ancestral estimates closer to those proposed by the SYM models. The results suggest that ancestral estimates at nodes close to the root are particularly sensitive to outgroup selection. However, we find that this is much less the case for nodes farther from the root. For instance, the analysis recovers the same likelihoods at node A under the ARD model, regardless of the outgroup selected (Fig. 3). At nodes D and F, the likelihoods differ strongly depending on the outgroup selected and can lead to very different conclusions regarding the absence or presence of plumage in these ancestors. The uncertainties calculated are higher under the ARD model, with the highest uncertainty occurring for Outgroup 4 (i.e., the tree with an extant outgroup).

As detailed earlier (see the Materials and Methods section), trees resulting from this experiment can be compared using a procedure similar to LOOCV. The results suggest that the outgroup has a limited influence on the average error in estimating tip states within the outgroup (Table 4). With an ARD transition rate model, the difference of mean error between the highest error (associated with Outgroup 1) and the lowest error (Outgroup 3) is of about 0.01. This is even lower with an SYM model. Thus, transition rate model selection seems to have substantially more influence on the results than outgroup selection. Indeed, although Outgroup 4 has the lowest error in the case of the ER and SYM models, it is only between 0.003 and 0.006 lower than Outgroup 3. To further discriminate between the different outgroup choices, we looked at the amount of information the model and tree assign to the Avemetatarsalia node (i.e., the most recent common ancestor of the ingroup). We find that Outgroups 3 and 4 consistently provide more information than Outgroups 1 and 2 (Table 5). In the case of the ARD model, Outgroup 3 provides significantly more information, nearly twice as much as Outgroup 4. Therefore, we are inclined to favor Outgroup 3 for conducting ASE analyses with this data set.

**Table 4.** Mean error of the model in estimating the tip states of the tree ingroup (obtained from a procedure similar to LOOCV; see the Materials and Methods section for details)

	ER	SYM	ARD
Outgroup 1	0.4107	0.3679	0.3661
Outgroup 2	0.4103	0.3676	0.3635
Outgroup 3	0.4093	0.3670	0.3591
Outgroup 4	0.4033	0.3639	0.3611

Note: Coding 3 is used here.

**Table 5.** Mutual information (in bits) assigned by the model and tips to the Avemetatarsalia node

	ER	SYM	ARD
Outgroup 1	2.4633	2.4396	0.3328
Outgroup 2	2.4925	2.4660	0.4105
Outgroup 3	2.5549	2.5359	1.1899
Outgroup 4	2.5369	2.5254	0.6497

Note: Coding 3 is used here.

### Experiment 2: Effect of the Time-Scaling Method

Under the equal timePaleoPhy method, internal nodes are distributed somewhat regularly along the time axis. The overall variation in branch lengths appears lower than in the other trees. Compared with the equal timePaleoPhy method, the mbl method leads to a tree with longer internal branches and shorter terminal branches. The equal DatePhylo method appears intermediate between the other two methods described. Some internal areas of the tree display a high degree of similarity with the equal timePaleoPhy tree. However, there are some very short internal branches, as in the mbl method. Both the “equal datePhylo” and the “mbl” methods infer the root (avemetatarsalian node) at a younger age than the “equal tpp” method.

**Figure 4.**
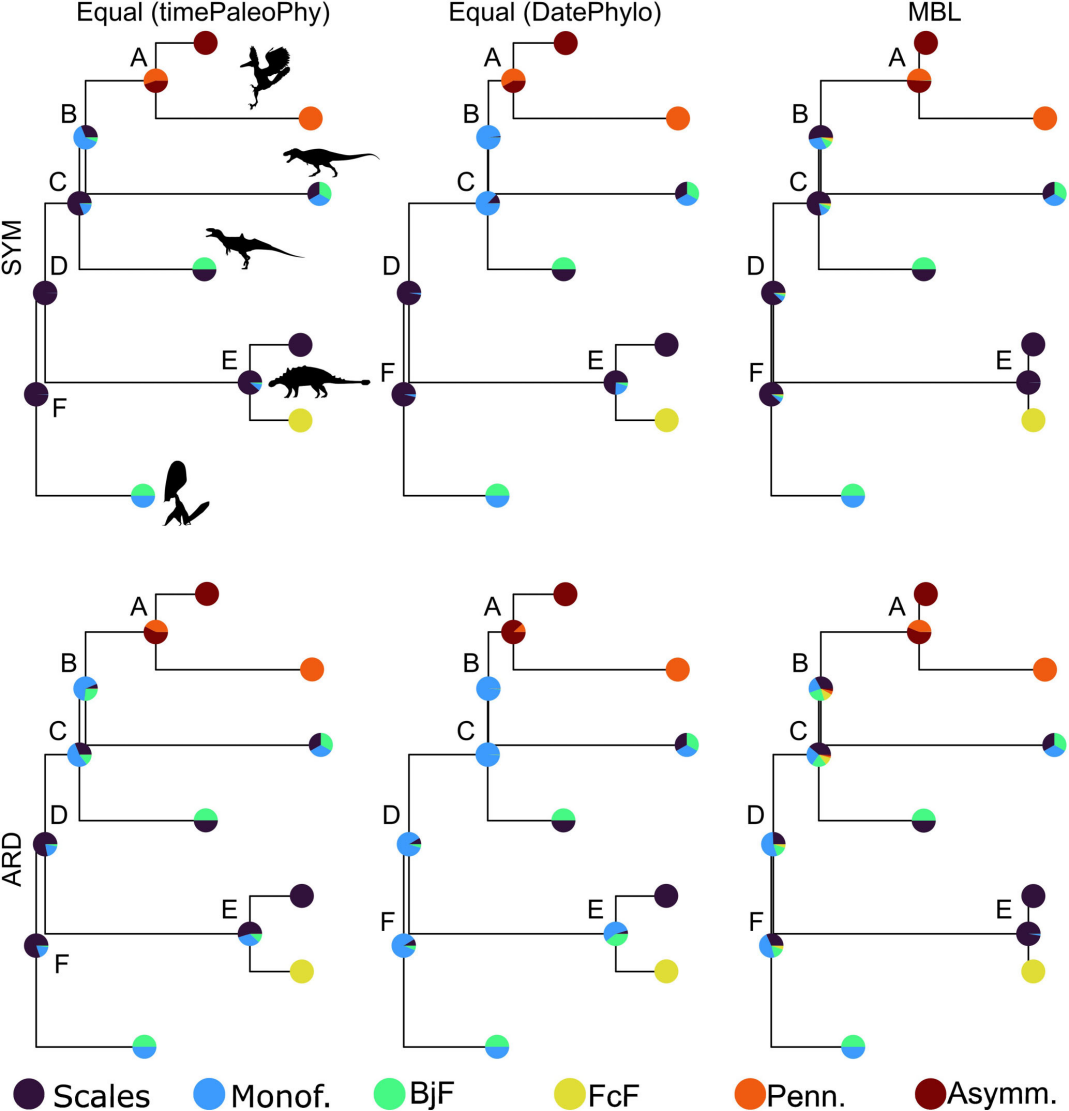
Comparison of the ancestral state likelihoods for three different trees resulting from two a posteriori time-scaling methods and two distinct packages (timePaleoPhy and DatePhylo). A subset tree with only six nodes is plotted to facilitate the comparison. Node labels and abbreviations in legend as in Figure 3. Silhouettes from PhyloPic.org. Attributions detailed in Figures 1 and 3.

Our results show that time-scaling method has a large impact on the results of our ASEs (Fig. 4). Ancestral likelihoods at nodes where the model is uncertain appear to be particularly sensitive to the time-scaling method used. Under the SYM model, there is a much higher likelihood of presence of feathers at nodes B and C with an equal DatePhylo tree than with an equal timePaleoPhy tree. AICc values suggest that the equal timePaleoPhy scaling method should be preferred in this case (Table 6).

**Table 6.** Raw uncertainty calculated for each tree obtained from different time-scaling methods

	SYM		ARD	
	Uncertainty (%)	AICc	Uncertainty (%)	AICc
Equal (DatePhylo)	13.61	220.40	9.74	254.69
Equal (timePaleoPhy)	14.66	207.76	17.41	248.26
mbl	32.38	241.80	32.46	270.19

Note: Coding 3 is used here.

### Experiment 3: Effect of the Coding Strategy

**Figure 5.**
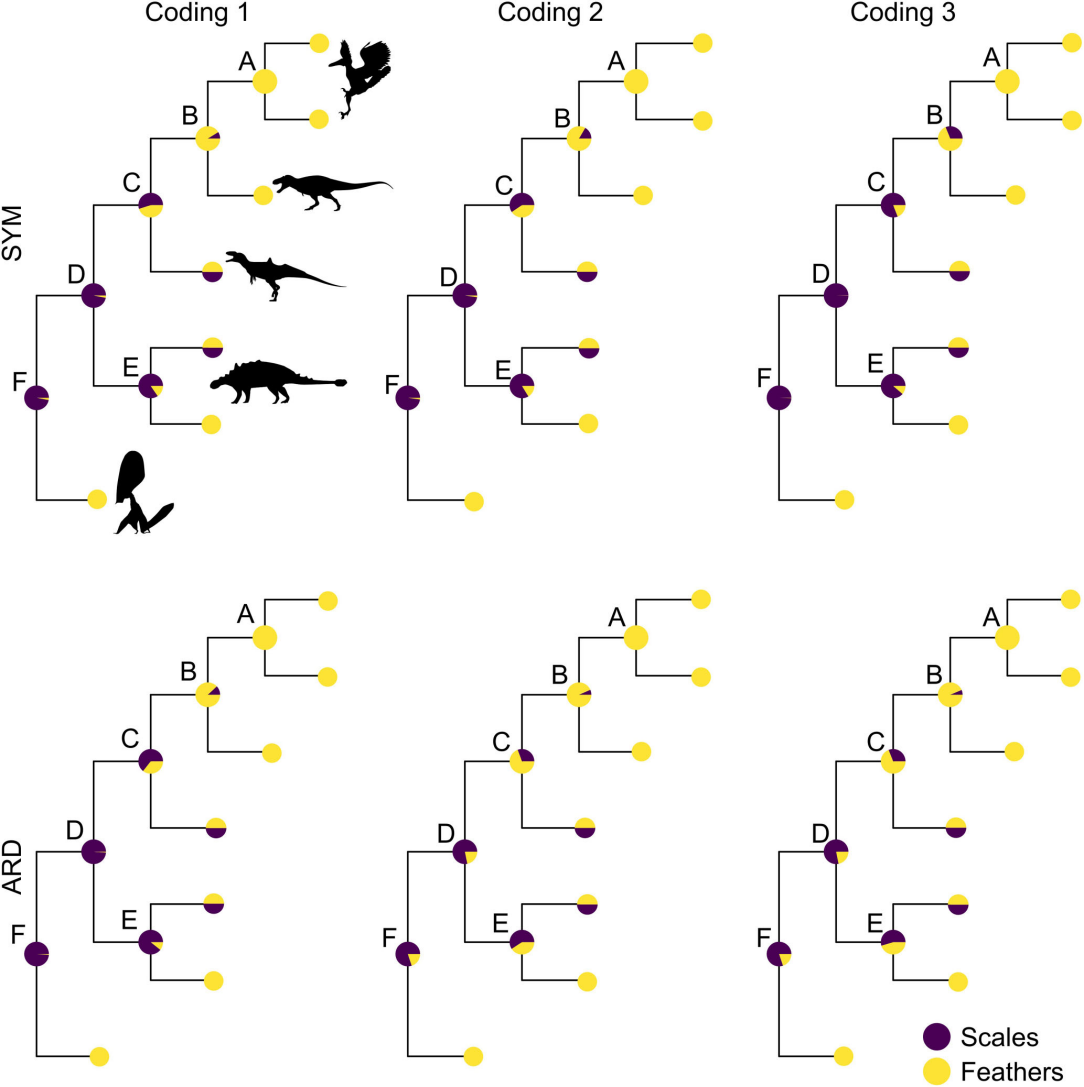
Comparison of the ancestral state likelihoods obtained for three different coding strategies. A subset tree with only six nodes is plotted to facilitate the comparison. For each coding strategy, the likelihoods of absence and presence of plumage are displayed through the aggregation of the likelihoods of the five feather morphotype states. This is effectively reducing Coding strategies 2 and 3 to Coding strategy 1, so that all the results can be compared. Node labels: A = most recent common ancestor (MRCA) of *Rahonavis* and *Sapeornis*; B = Coelurosauria; C = Tetanurae; D = MRCA of *Kulindadromeus* and *Psittacosaurus*; E = Dinosauria; F = Avemetatarsalia. Silhouettes from PhyloPic.org. Attributions detailed in Figures 1 and 3.

We find a positive correlation between uncertainty of ancestral state estimates and number of character states. Raw uncertainty is the lowest for the simplest coding strategy (Coding 1) and highest for the most granular coding strategy (Coding 3) (Table 7). Although all three coding strategies vary in terms of their uncertainty, they do not produce substantively different ancestral estimates with respect to the origin of feathers. This can be seen clearly if the likelihoods of different feather morphotypes are aggregated into a single state, equivalent to Coding scheme 1. All coding schemes estimate feather absence as the most likely state at the Avemetatarsalia and Dinosauria nodes (nodes D and F; Fig. 5). Under the SYM model, nodes B and C, farther from the root, the likelihood of feather absence seems to increase as the coding strategy becomes more granular. A somewhat opposite pattern is observed under ARD. At node E, increasing the number of states increases the likelihood of presence of plumage.

**Table 7.** Raw uncertainty (%) of the ancestral likelihoods calculated for each coding strategy tested

	ER	SYM	ARD
Coding 1	4.24	4.24	3.32
Coding 2	4.63	5.40	8.28
Coding 3	10.11	14.66	17.41

### Experiment 4: Effect of the Model

**Figure 6.**
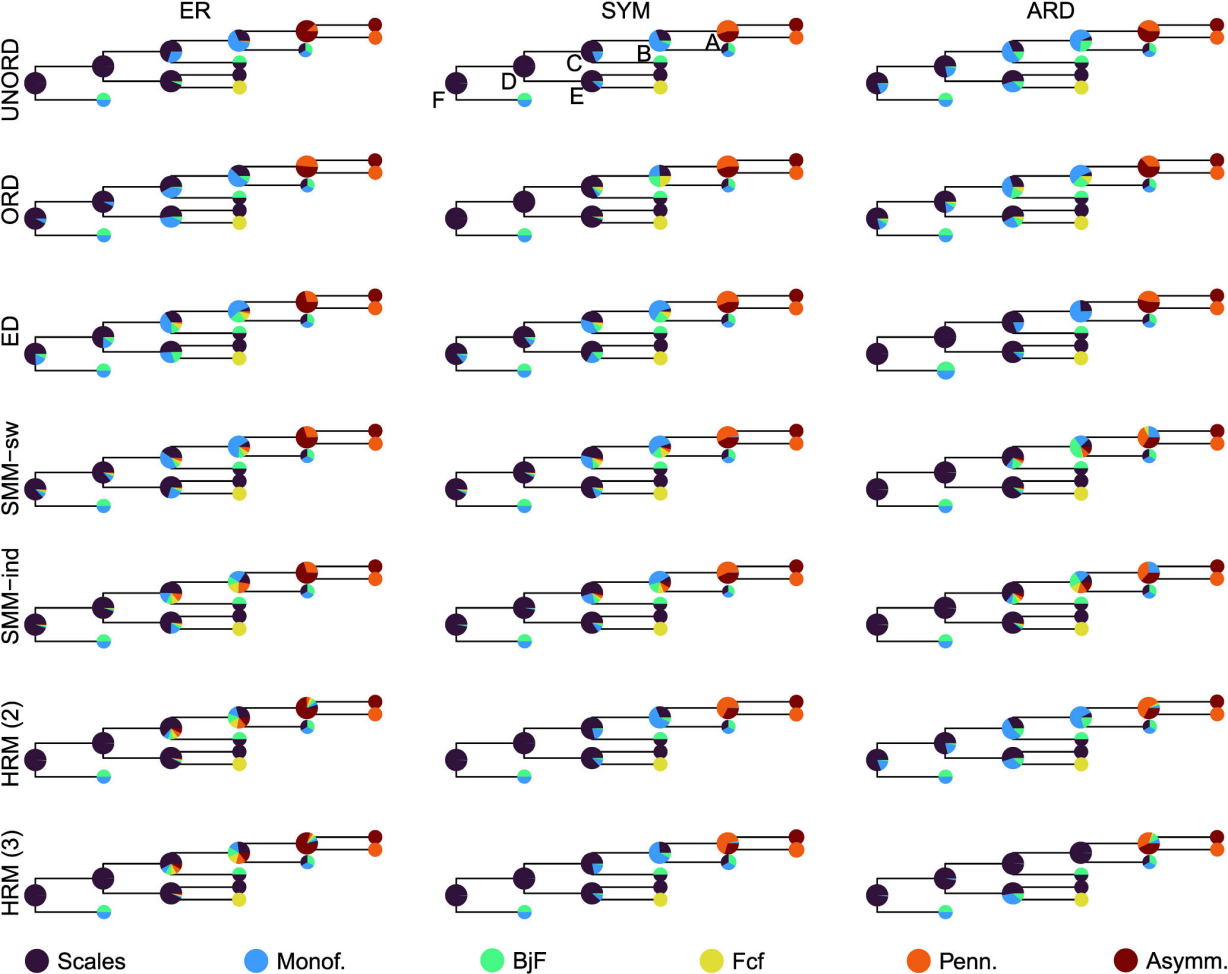
Comparison of the ancestral state likelihoods for seven different models under ER, SYM, and ARD. A subset tree with only six nodes is plotted to facilitate the comparison. Node labels and abbreviations as in Figure 3.

We estimated ancestral states under three transition models (ER, SYM, and ARD) and seven different Markov model architectures (unordered, ordered, ED, SMM-ind, SMM-sw, HRM(2), and HRM(3)) resulting in 21 models (Supplementary Table S3). The results of these analyses are summarized using a simplified version of the phylogenetic tree in Figure 6.

All 21 models tested support the hypothesis of several independent origins of feathers throughout their evolutionary history. The presence of scales in the avemetatarsalian ancestor of dinosaurs and pterosaurs is inferred with likelihoods ranging from about 76% (ER ED) to more than 99% (ER UNORD). Most of the models also infer the absence of feathers at node C, although a few suggest their possible presence (e.g., ER ORD, ER ED, ARD UNORD). The hidden Markov models (SMM and HRM) tend to be uncertain regarding the ancestral state at node B, and to some extant at node C, as well.

Using AIC for model selection, the ARD ORD model is best at explaining the data observed. However, the model returning results with the lowest uncertainty is ER UNORD. Ordered models are associated with a higher uncertainty of the results. Here, the effect is strongest under an ER model where the uncertainty nearly doubles with ordering. Among all the 21 models tested, the lowest uncertainty of results is achieved with the ER unordered model, which is the simplest model tested with only a single parameter to estimate. Its AIC is among the highest (indicating a poor fit of the model to the data) observed in this study.

The SMM models are associated with very high uncertainties of results under ARD. This uncertainty is much lower under the ER and SYM models.

In the case of the HRM models, the uncertainty does not seem to be correlated with the number of rate categories, or even the transition rate model used. In fact, the uncertainty is even higher under ER than SYM or ARD, which is not what is observed with the other models.

### Tree/Model Combinations

In the previous section, we tested the models using the equal timePaleoPhy tree. However, different trees may be favored under different models. To test whether this is the case here, we ran additional analyses with the equal DatePhylo and mbl trees, thus resulting in 63 tree/model combinations (Supplementary Table S4). Seven combinations inferred the presence of plumage at the avemetatarsalian node. Except for two combinations, these are associated with a high uncertainty of the ancestral state estimates. The favored model is a function of the metrics used: AIC/AICc (ARD ORD, mbl tree), or LOOCV mean error (18 models have errors within 10% of the lowest error, thus indicating comparable generalizability with the lowest error model). We calculated Akaike weights (Akaike 1978) and followed a similar approach for the LOOCV mean error (see Model Averaging in the Materials and Methods section). The ancestral likelihoods resulting from this model averaging approach, whether they rely exclusively on Akaike weights (Supplementary Fig. S5) or LOOCV mean error weights, do not lead to different evolutionary interpretations.

### Cross-validation Approach

**Figure 7.**
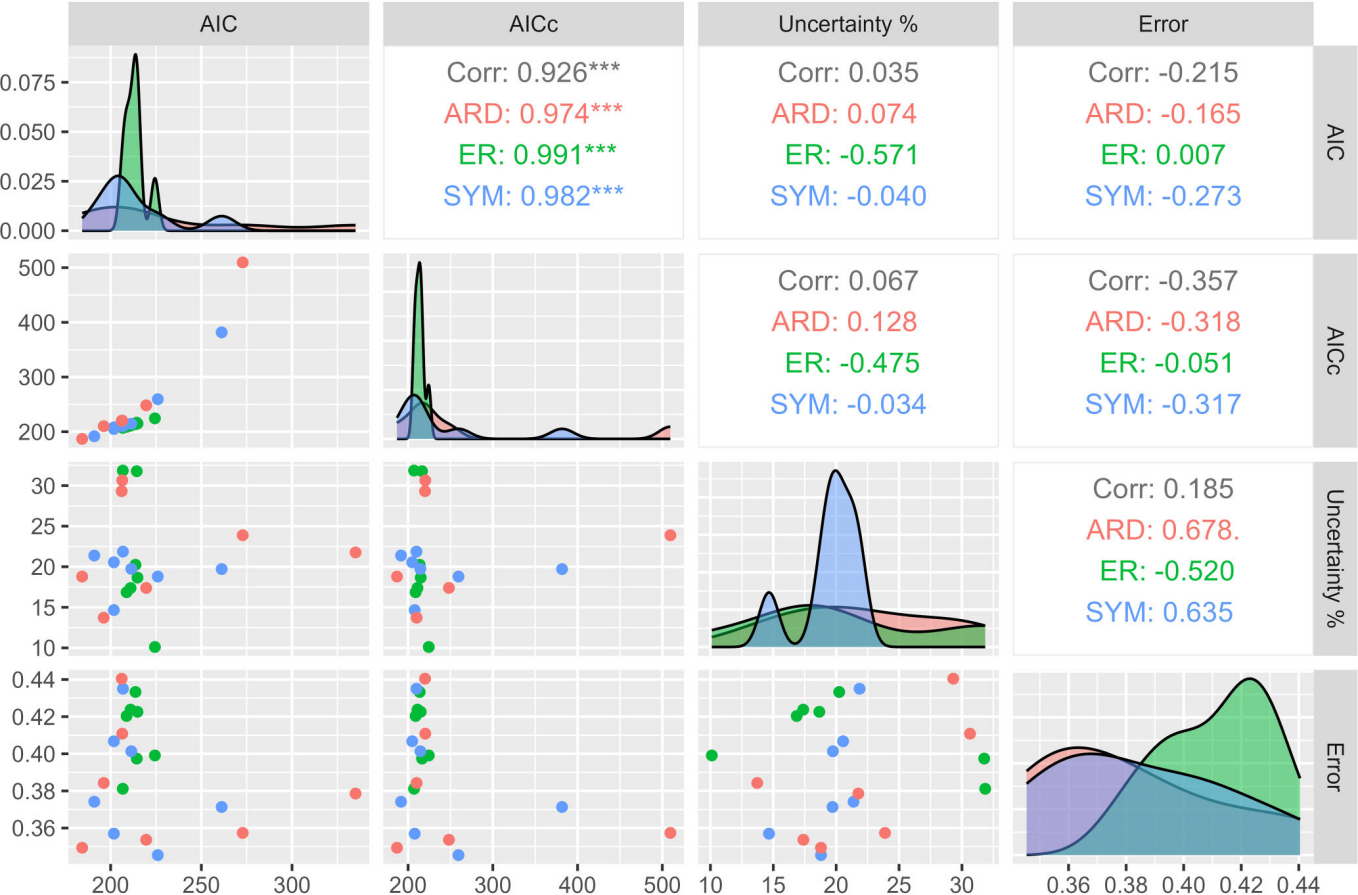
Comparison of the 21 models tested in this study using the AIC, AICc, uncertainty, and LOOCV mean error of the models. Density plots in the diagonal show the distribution of ER, SYM, and ARD data for each of the variables. Pearson correlation coefficients are shown in upper panels (on the right side of the diagonal), whereas scatter plots are located in lower panels (left side of the diagonal).

The LOOCV results (Supplementary Table S5) show that the mean error of the models ranges from approximately 0.33 to 0.45. Some models associated with high AIC and AICc values (e.g., SYM HRM 2 with an mbl tree) have in fact the lowest LOOCV mean errors out of the models tested (around 0.34). ARD HRM 3 represents a particular case though: the number of parameters of this model equals the number of tips, resulting in very low negative values of the AICc. However, no fewer than 19 models have LOOCV errors between 0.333 and 0.366. ARD ORD with an mbl tree is among these models and is also associated with the lowest AIC and AICc (when ARD HRM 3 is excluded). There is little correlation between AIC and LOOCV mean error (Fig. 7). Furthermore, information and uncertainty do not seem to be correlated with LOOCV mean error. Some models with low information have either low mean errors (ARD HRM models with an equal DatePhylo tree) or high mean errors (e.g., ARD SMM models with an equal DatePhylo tree). A similar observation can be made with the uncertainty metric. Models associated with low uncertainties, such as the ER UNORD model, also have high LOOCV mean errors.

## Discussion

### Outgroup Importance

Our results show that choice of outgroup has little influence on ancestral state estimates under the SYM model. The ancestral likelihoods appear identical across the analyses performed with four different outgroups, but this is not the case under the ARD model. Our analyses show that increasing the number of taxa comprising the outgroup changes the ancestral likelihoods at key nodes. Although the analyses performed with an outgroup comprising a single taxon or two taxa estimate the presence of feathers at the root of the tree (i.e., avemetatarsalian node), analyses with an outgroup comprising three taxa estimate a higher likelihood of absence of any plumage at that node. This implies that, in some cases, interpretations surrounding the evolution of a trait can depend on the outgroup selected. For instance, Yang et al. (2019) and Cincotta et al. (2022) proposed the hypothesis of a single point of origin of feathers around 240 Ma at the avemetatarsalian node, based on maximum likelihood methods. In both studies, an ARD model was selected, and an outgroup formed of a single taxon (Yang et al. 2019) was used, or the analysis was conducted without an outgroup (Cincotta et al. 2022). Including several outgroups may allow estimation of the ancestral states with a higher confidence at some nodes close to the root. However, adding numerous outgroups may also involve a risk of biasing the rate estimation. Such issues are of particular importance in the case of ASE analyses of feather evolution. As mentioned earlier, previous studies have led to contradictory results regarding the origin(s) of feathers. Although some analyses recovered the presence of filamentous integument in the ancestor of pterosaurs and dinosaurs (Avemetatarsalia), others found several independent origins within Pterosauria and Dinosauria. The avemetatarsalian node is close to the root of the phylogenetic tree and, therefore, ancestral state estimates at this node are potentially very sensitive to outgroup selection. We suggest selecting multiple outgroup taxa representing a diversity of lineages (within a more inclusive clade including the ingroup) and assessing the effect of outgroup selection on the analyses by exploring how the ancestral estimates of basal nodes vary with alternative outgroups.

### Time-Scaling Importance

Several studies have shown that ASE is sensitive to branch lengths and thus to the a posteriori time-scaling method used (e.g., Bapst 2014; Bapst and Hopkins 2017). Our results support this assertion. The time-scaling methods impact the position of the internal nodes along the time axis and the branch lengths. The same model can lead to different results when various trees are used (see Supplementary Table S4). The equal timePaleoPhy tree is characterized by internal nodes more evenly distributed through time than the other two methods tested. In the equal DatePhylo tree, some branches are elongated or shortened, resulting in an apparent shift toward the tips of a series of internal nodes. This is also the case for the mbl tree but is even more pronounced. Within Ornithischia some internal branches are long, and multiple nodes are placed much closer to the tips than in the other trees. We recommend testing different combinations of models and time-scaling methods. Leave-one-out cross-validation can then be used to select the best combination. Prior knowledge of the biological processes studied may also guide the selection of the best tree and model combination. In some cases, external evidence from other fields might be the only way to select ASE results (Skinner 2010). Model averaging can also be used to incorporate the uncertainty from different time-scaling methods. It is worth keeping in mind that topological uncertainty is as relevant as the tree-scaling method used: the branch lengths and distribution of tip states affect the ASE results.

### Coding Strategy

All the coding strategies tested retained sufficient historical information on the trait studied. Increasing the number of states increased the uncertainty of the results (Table 5). Under both SYM and ARD, the coding strategy seems to affect the estimated likelihoods of presence of plumage at some nodes (Fig. 5). However, the impact remains limited, and the overall interpretation of the evolutionary history did not change. These results suggest that coding strategies are less of a concern than we had feared; the definitions of states and their granularity do not necessarily lead to different macroevolutionary inferences in the case of feather evolution. It remains to be determined whether this represents a general pattern, so we recommend researchers exercise caution when considering alternate coding strategies in ASE studies. We suggest that the impact of such strategies be investigated using an approach similar to the one employed in this study.

### Model Selection

AIC/AICc scores can be used to evaluate the relative fitness of a model. However, rather than simply evaluating how well the models fit the data (i.e., the tip states), our aim is to find the model that most accurately infers ancestral states. LOOCV based on reconstruction error provides a direct assessment of a model’s generalizability—that is, its ability to accurately predict unseen data. As such, we consider this approach the most appropriate for evaluating how well a model estimates ancestral states. Consequently, we used LOOCV with error-based metrics as the model selection criterion in this context. Our results reveal little correlation between AIC/AICc and LOOCV mean error, suggesting no clear relationship between model fit and generalizability. This discrepancy is likely driven by the limited data set size and the information content of the tip states. Because LOOCV is asymptotically equivalent to AIC (Stone 1977), we expect that with a sufficiently large number of informative tips, information criteria, and LOOCV error would converge. However, given the small data sets typical of many empirical ancestral state reconstructions, particularly in paleontological studies, it is important to emphasize that model fit does not necessarily imply model generalizability.

The LOOCV results suggest that the most generalizable models are the SYM and ARD models (ordered and unordered), as well as the SYM and ARD HRM models. The SMM are associated with the highest LOOCV mean errors: between approximately 0.38 and 0.45. SMM involve many more free parameters than standard Markov models, so are strongly data dependent (Tarasov 2019). Beaulieu et al. (2013) found that trees with a minimum of 60–120 taxa are required so that HRM can be used in ASE. However, the ER models (unordered and ordered) are similarly associated with high LOOCV mean errors (ranging from about 0.39 to 0.44). This implies that these models are strongly biased: with a single parameter, they are too simple to model feather evolution accurately and explain the data observed.

### Implications for Feather Evolution

**Figure 8.**
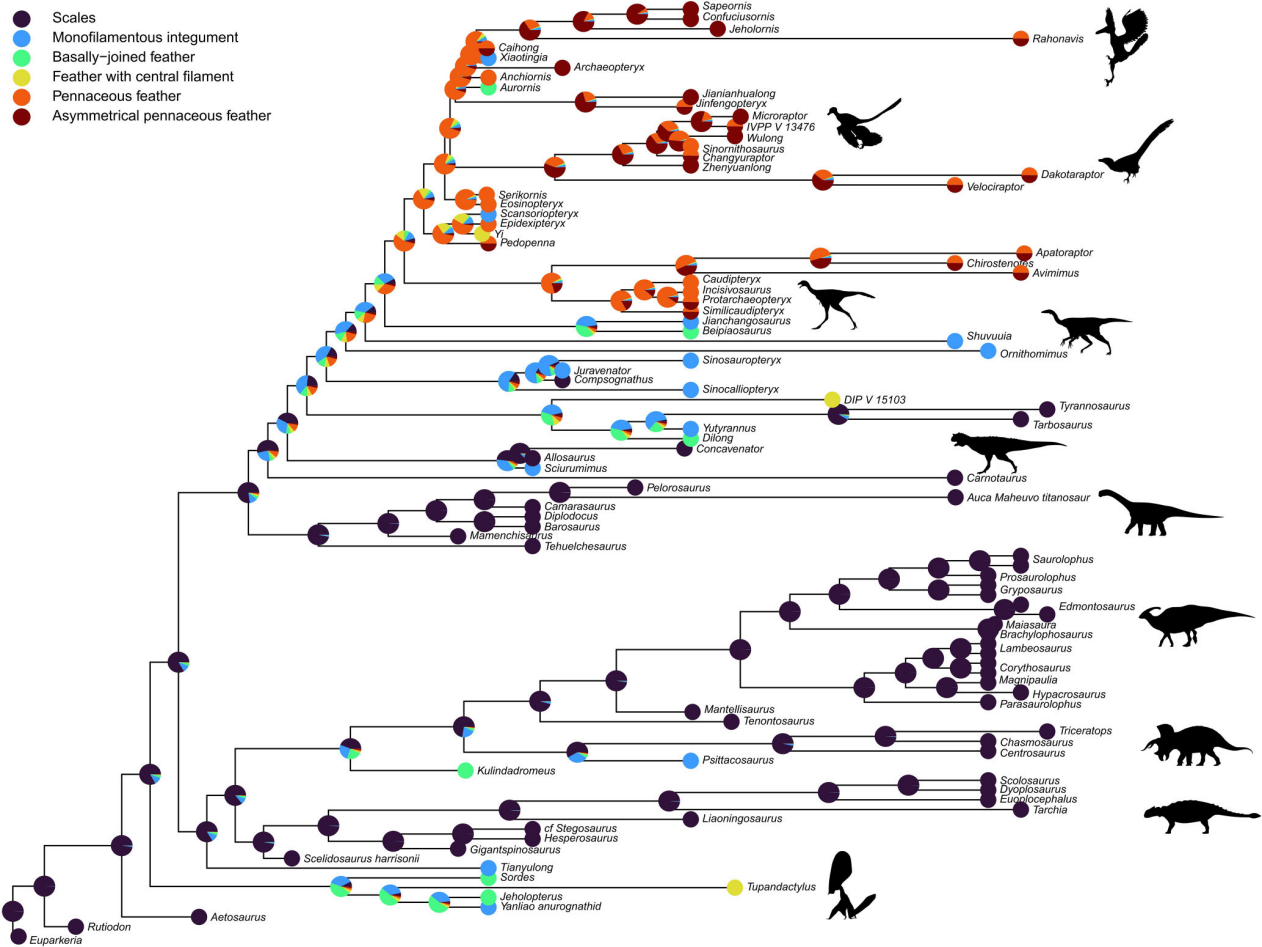
Average of the 63 model/tree combinations using weightings based on the error of the models. The ancestral likelihoods are plotted on the equal (timePaleoPhy) tree. Silhouettes from PhyloPic.org. Attributions detailed in Figure 1.

Most of the models tested support the hypothesis of feathers originating several times throughout their evolutionary history. Only seven model/tree combinations out of 63 tested infer the presence of feathers at the avemetatarsalian node. The ARD ordered models selected based on the AIC suggest a minimum of three independent points of feather origin within Pterosauria, Saurischia, and Ornithischia. A similar observation can be made with an average of the models using weightings based on the LOOCV mean error of the models (Fig. 8). If that is the case, this would correspond to a late origin with filaments homologous to modern bird feathers evolving between 190 and 212 Ma, as opposed to an early origin of feathers suggested by the results of previous ASE analyses (Yang et al. 2019; Cincotta et al. 2022).

Pennaceous feather morphotypes have been reported on the forelimb and tail, from basal and more derived paravian taxa, and from Oviraptorosauria. Our analyses support the hypothesis that pennaceous feathers were inherited from a common ancestor of Paraves and Oviraptorosauria, in the Lower Jurassic (∼185 Ma). Based on the presence of quill knobs, pennaceous feathers have been inferred in a specimen of *Ornithomimus* (Zelenitsky et al. 2012). However, the preserved morphology would suggest rather that these are broad monofilamentous feathers (Xu 2020). Feather specialization with short coverts overlapping longer flight feathers is observed in Dromaeosauridae (e.g., *Microraptor, Changyuraptor*, and *Zhenyuanlong*) and in basal avian taxa (*Archaeopteryx, Jeholornis, Sapeornis*, and *Confuciusornis*). The asymmetry of flight feathers follows a similar evolutionary pattern: asymmetrical feathers were observed in dromaeosaurid dinosaurs (e.g., *Zhenyuanlong, Wulong*, and *Microraptor*) and avian taxa. Because the forelimb plumage is not preserved in troodontid specimens, it is unclear whether feather specialization and asymmetry occurred separately in Dromaeosauridae and Aves (i.e., convergent evolution) or was inherited from a common ancestor.

Although these results seem to refute the hypothesis of feathers being a synapomorphy of Pterosauria and Dinosauria, further investigation is required to clarify the early evolutionary history of feathers. Some of the models tested in this study, in particular the HMMs, may perform better with a tree comprising additional tips. Further, a major limitation of recent studies, including this one, is related to the way the plumage information is coded. Only the most developmentally complex morphotype is considered even when several are observed within a taxon. A prerequisite for significantly improving future ASE analyses may involve developing new ways to include such information. A possible solution requires using models in which polymorphic states are included and treated as intermediate between the corresponding monomorphic conditions.

## Conclusions

Feather evolution served as an ideal case study to explore different statistical approaches in ASE of complex characters. We tested the effect of different outgroups, branch lengths, coding strategies, and models on our results. All these factors influenced the end results on their own or in association. Among the models tested, ARD ORD with an mbl tree is associated with the lowest AIC and AICc suggesting the best model fit. A cross-validation approach revealed that this model is the second one with the lowest LOOCV error. However, our results do not show a strong correlation between AIC or AICc and LOOCV error, indicating that, for our data set, model fitness does not predict model generalizability. Nonetheless, both sets of results support the hypothesis of several independent origins of feathers.

Ultimately these results inform best practices to adopt when modeling complex discrete characters. It is critical to carefully establish the appropriate coding strategy, allowing to provide the maximum information on the processes studied. The outgroup selection and branch lengths must be carefully considered as well. Finally, analyses should be conducted with several statistical methods.

## Supplementary Material

syaf063_Supplemental_File

## Data Availability

The data underlying this article are available in the Dryad Digital Repository at: https://doiorg/10.5061/dryad.4tmpg4fq3
