# Supplementary material for: Estimating Ancestral States of Complex Characters: A Case Study on the Evolution of Feathers
Source: Syst Biol. 2025 Sep 13;75(2):296–312. doi: 10.1093/sysbio/syaf063 (PMC13016762; doi:10.1093/sysbio/syaf063)
Supplement: syaf063_Supplemental_File [file syaf063_supplemental_file.pdf]

SUPPLEMENTAL INFORMATION

Estimating ancestral states of complex characters: a case study on the evolution of feathers

Pierre Cockx, Michael J. Benton, Joseph N. Keating

Table of Contents

Figure S1 1
Table S1 2
Table S2 2
Table S3 2
Figure S2 3
Figure S3 4
Figure S4 4
Figure S5 5
Table S4 6
Table S5 7
Table S6 8
SUPPLEMENTARY TEXT 10
SUPPLEMENTARY REFERENCES 11

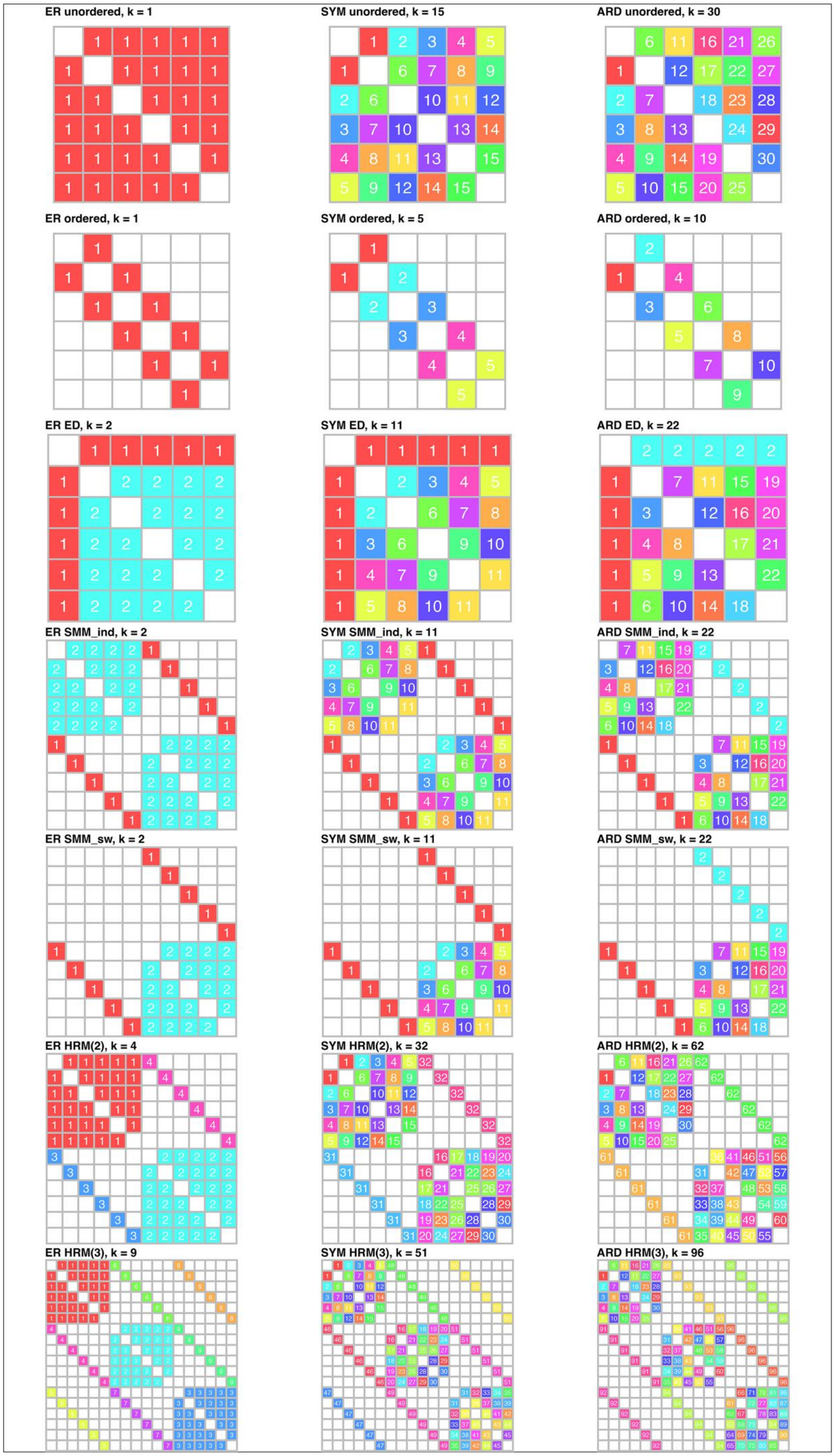

FIGURE S1. Diagrammatic representations of the 21 Markov Models tested in Experiment 4. k = number of rate parameters.

	ER		SYM		ARD	
	Info.	Uncert. %	Info.	Uncert. %	Info.	Uncert. %
Outgroup 1	206.59	10.46	201.62	15.07	86.67	18.11
Outgroup 2	201.62	10.30	209.35	14.88	92.63	18.14
Outgroup 3	212.27	10.11	207.14	14.66	141.60	17.40
Outgroup 4	213.56	09.71	207.41	14.53	125.11	19.56

TABLE S1. Outgroup effect.

	ER			SYM			ARD		
	Info	Uncert. %	AIC	Info.	Uncert. %	AIC	Info.	Uncert. %	AIC
Equal (DatePhylo)	214.84	8.74	240.31	211.03	13.61	214.40	87.49	9.74	226.07
Equal (timePaleoPhy)	212.27	10.11	224.4	207.14	14.66	201.76	141.57	17.41	219.64
mbl	185.94	20.21	260.74	170.05	32.38	235.80	115.70	32.46	241.57

TABLE S2. Time-scaling effect.

	**ER**			**SYM**			**ARD**		
	Uncert.	Info.	AIC	Uncert.	Info.	AIC	Uncert.	Info.	AIC
**UNORD**	0.084	212.27	224.4	0.122	207.14	201.8	0.145	141.60	219.6
**ORD**	0.169	190.90	213.7	0.178	194.52	191.0	0.157	115.99	184.3
**ED**	0.155	102.49	214.8	0.164	107.70	211.4	0.114	172.00	196.2
**SMM-sw**	0.145	260.88	211.0	0.182	209.06	206.8	0.255	101.62	206.4
**SMM-[illegible]**	0.140	236.97	208.8	0.171	194.31	201.8	0.244	144.49	206.1
**HRM (2)**	0.265	200.70	206.7	0.157	201.55	226.0	0.199	134.93	272.8
**HRM (3)**	0.265	204.85	214.4	0.164	193.65	261.2	0.181	145.45	335.0

TABLE S3. Information (in bits), uncertainty and AICc calculated for each model tested in Experiment 4. Abbreviation: Info.= Information (bits); Uncert.=Uncertainty.

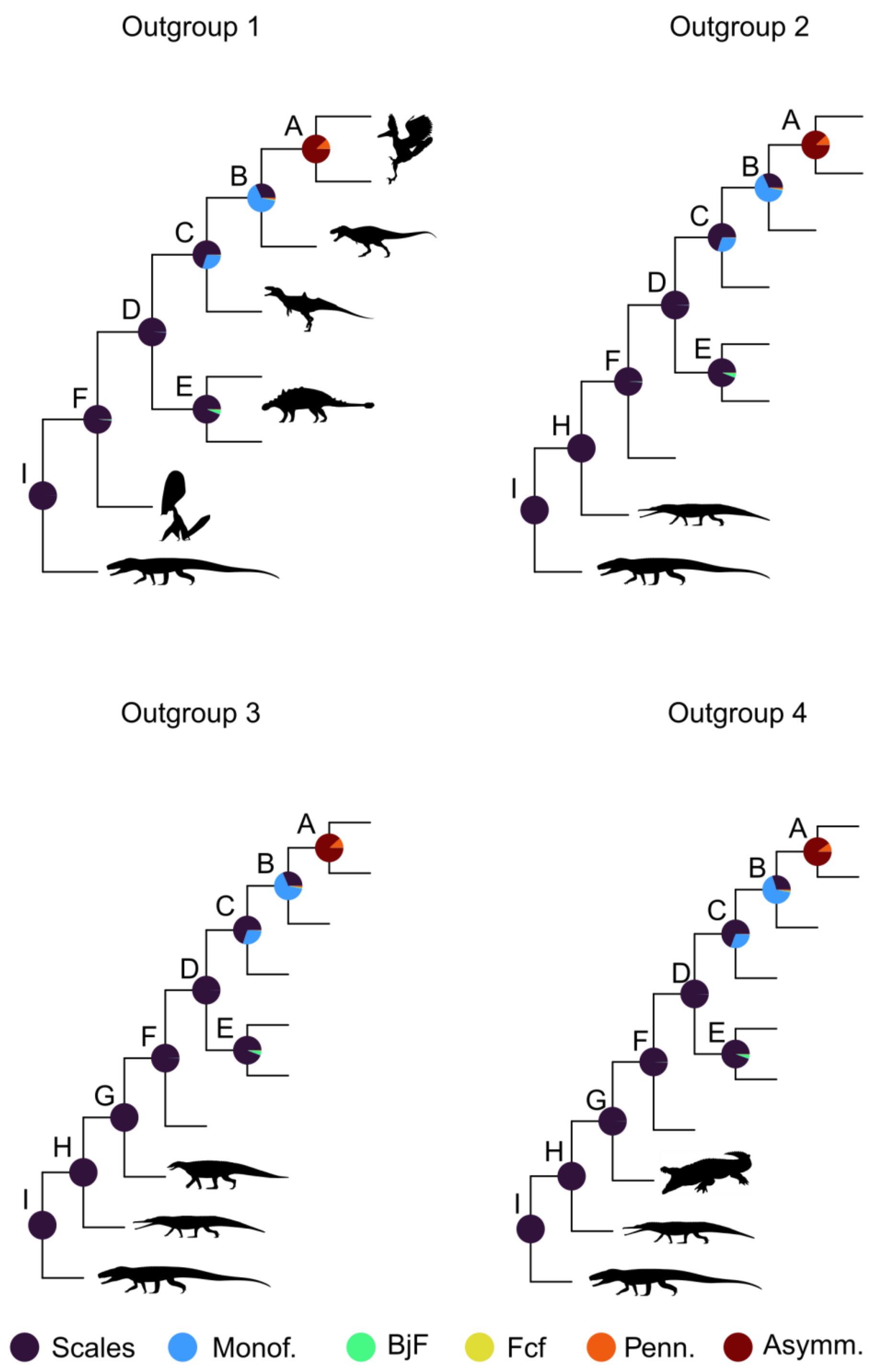

FIGURE S2. Comparison of the ancestral state likelihoods under an ER transition rate model, for four different outgroups. Silhouettes from PhyloPic.org. Attributions are detailed in the captions of Figures 1 and 3 of the main text.

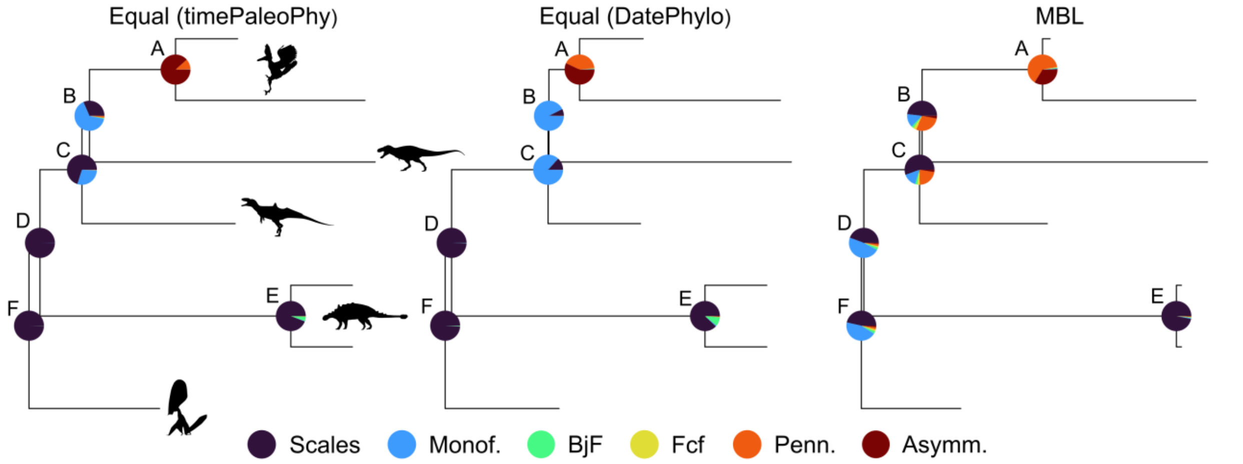

FIGURE S3. Comparison of the ancestral state likelihoods under an ER transition rate model, for three different trees resulting from two a posteriori time-scaling methods and two distinct packages (timePaleoPhy and DatePhylo). Silhouettes from PhyloPic.org. Attributions are detailed in the captions of Figures 1 and 3 of the main text.

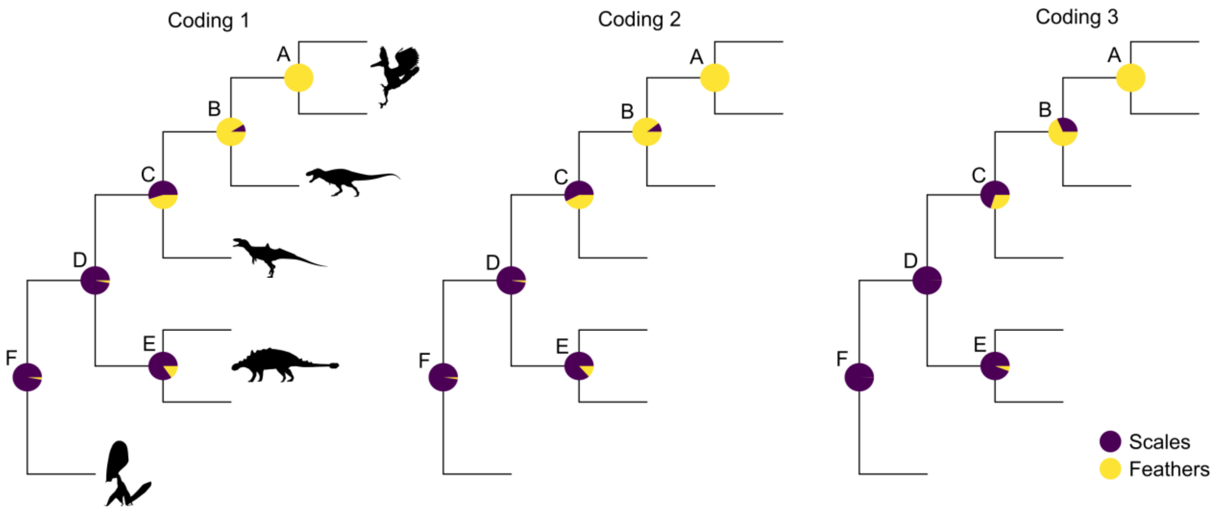

FIGURE S4. Comparison of the ancestral state likelihoods under an ER transition rate model, for three different coding strategies. Silhouettes from PhyloPic.org. Attributions are detailed in the captions of Figures 1 and 3 of the main text.

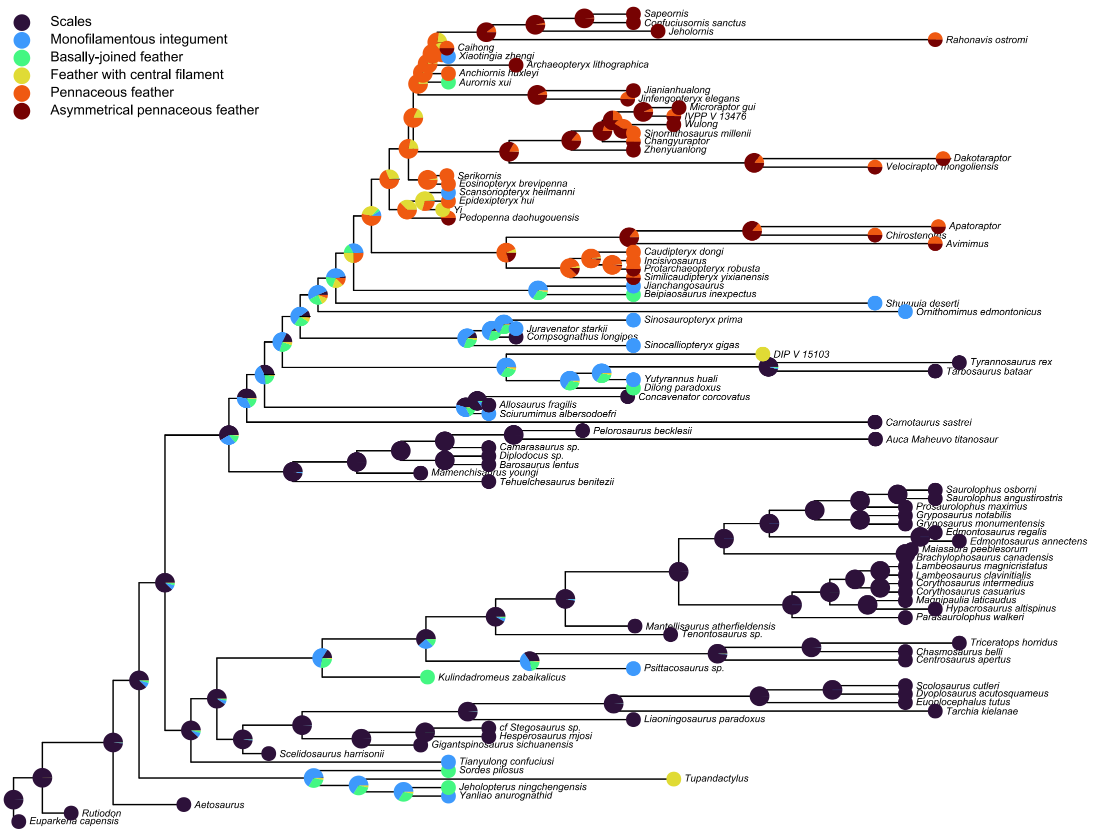

FIGURE S5. Average of the 63 model/tree combinations using weightings based on AIC. The ancestral likelihoods are plotted on the equal (timePaleoPhy) tree.

	ER				SYM				ARD			
	Uncert %	AIC	AICc	Avem.	Uncert %	AIC	AICc	Avem.	Uncert %	AIC	AICc	Avem.
**UNORD**												
Equal tpp	10.11	224.4	224.4	0.997	14.66	201.8	207.8	0.995	17.39	219.6	248.2	0.803
Equal dp	8.74	240.3	240.3	0.991	13.61	214.4	220.4	0.962	9.74	226.1	254.7	0.087
mbl	10.49	227.9	227.9	0.992	14.83	201.5	207.5	0.993	18.76	218.8	247.4	0.930
**ORD**												
Equal tpp	20.24	213.7	213.7	0.925	21.38	191.0	191.7	0.998	18.79	184.3	186.9	0.790
Equal dp	17.85	245.3	245.3	0.870	25.40	206.2	206.9	0.990	20.95	188.5	191.1	0.054
mbl	21.70	205.1	205.1	0.907	18.82	190.3	191.0	0.995	19.97	181.6	184.2	0.926
**ED**												
Equal tpp	18.66	214.8	214.9	0.758	19.74	211.4	214.5	0.858	13.72	196.2	210.1	0.997
Equal dp	18.91	234.3	234.4	0.228	24.02	228.4	231.5	0.336	13.53	211.2	225.1	0.952
mbl	16.20	220.5	220.6	0.356	19.49	220.7	223.8	0.336	17.39	200.1	214.0	0.995
**SMM-sw**												
Equal tpp	17.38	211.0	211.1	0.871	21.86	206.8	209.9	0.925	30.64	206.4	220.3	0.989
Equal dp	20.41	227.6	227.7	0.656	25.64	217.7	220.8	0.813	37.28	205.3	219.2	0.830
mbl	16.15	214.5	214.6	0.818	24.65	212.5	215.6	0.824	32.22	203.5	217.4	0.950
**SMM-ind**												
Equal tpp	16.86	208.8	208.9	0.950	20.55	201.8	204.9	0.970	29.31	206.1	220.0	0.989
Equal dp	17.03	225.2	225.3	0.878	22.48	216.0	219.1	0.882	36.95	205.3	219.2	0.830
mbl	14.11	212.3	212.4	0.927	21.05	208.5	211.6	0.915	30.91	203.3	217.2	0.956
**HRM (2)**												
Equal tpp	31.85	206.7	207.1	0.991	18.79	226.0	259.5	0.993	23.88	272.8	509.5	0.806
Equal dp	25.11	204.1	204.5	0.961	18.59	225.4	258.9	0.975	13.95	266.3	503.0	0.078
mbl	33.69	206.1	206.5	0.971	19.80	222.3	255.8	0.868	18.36	267.0	503.7	0.964
**HRM (3)**												
Equal tpp	31.77	214.4	216.5	0.997	19.71	261.2	381.7	0.994	21.76	335.0	-	0.982
Equal dp	24.98	212.3	214.4	0.978	19.36	255.8	376.3	0.694	14.99	324.7	-	0.998
mbl	33.88	214.8	216.9	0.985	28.34	252.2	372.7	0.748	27.53	328.8	-	0.559

TABLE S4. Statistics of the 63 model/tree combinations tested. Abbreviations: Info. = Information; Uncert.=Uncertainty; Avem.= estimated likelihood of scales present at the avemetatarsalian node; tpp=timePaleoPhy; dp=DatePhylo. Yellow highlight: lowest AIC and AICc values. Red font: lowest likelihoods of scales.

	Equal tpp	Equal dp	mbl
ER UNORD	0.39909999	0.41934149	0.3908278
ER ORD	0.43327067	0.44325583	0.4143599
ER ED	0.42260090	0.44325583	0.4143599
SYM UNORD	0.35709636	0.35583714	0.33308197
SYM ORD	0.3748132	0.40676280	0.36410167
SYM ED	0.4013658	0.4202267	0.37239214
ARD UNORD	0.35173635	0.34668781	0.35199931
ARD ORD	0.34925913	0.35153896	0.34182526
ARD ED	0.38425777	0.37812650	0.38433802
ER SMM-ind	0.4203565	0.4440503	0.3875703
ER SMM-sw	0.4237796	0.4533903	0.3985766
SYM SMM-ind	0.4054037	0.4516672	0.4188037
SYM SMM-sw	0.4236432	0.4456051	0.4445315
ARD SMM-ind	0.4346315	0.4422652	0.4203093
ARD SMM-sw	0.4067073	0.4245235	0.3976298
ER HRM (2)	0.3805836	0.3909522	0.3746663
ER HRM (3)	0.3969093	0.3811961	0.3803824
SYM HRM (2)	0.3570135	0.3654605	0.3442252
SYM HRM (3)	0.3601266	0.3820500	0.3498191
ARD HRM (2)	0.3557437	0.3564497	0.3628823
ARD HRM (3)	0.3683926	0.3760562	0.3598471

TABLE S5. Mean error calculated for each of the tree-model combinations using a leave-one-out cross-validation (LOOCV) approach. Green highlight: lowest mean LOOCV error. Yellow highlight: mean LOOCV error within +10% of the lowest value.

**Taxon**	**References**
Ambopteryx	Wang et al. (2019)
Anchiornis	Hu et al. (2009)
Apataraptor	Funston and Currie (2016)
Archaeopteryx	Christiansen and Bonde (2004)
Aurornis	Godefroit et al. (2013)
Beipiaosaurus	Xu et al. (1999a, 2009)
Caihong	Hu et al. (2018)
Caudipteryx	Ji et al. (1998); Zhou and Wang (2000)
Changyuraptor	Han et al. (2014)
Chirostenotes	Funston and Currie (2021)
Confuciusornis	Hou et al. (1995); Zhou and Hou (1998); Chiappe et al. (1999); Foth (2012)
Dakotaraptor	DePalma et al. (2015)
Dilong	Xu et al. (2004)
DIP-V-15103	Xing et al. (2016)
Eosinopteryx	Godefroit et al. (2013)
Epidexipteryx	Zhang et al. (2008)
Incisivosaurus	Xu et al. (2010)
Jeholopterus	Wang et al. (2002); Kellner et al. (2010)
Jeholornis	O'Connor et al. (2013)
Jianchangosaurus	Pu et al. (2013)
Jinfengopteryx	Ji et al. (2005)
Juravenator	Bell and Hendrickx (2021)
Kulindadromeus	Godefroit et al. (2020)
Microraptor	Xu et al. (2003)
Ornithomimus	Zelenitsky et al. (2012); van der Reest et al. (2016)
Pedopenna	Xu and Zhang (2005)
Protarchaeopteryx	Ji and Ji (1997); Ji et al. (1998)
Psittacosaurus	Mayr et al. (2002, 2016); Bell et al. (2022)
Rahonavis	Forster et al. (1998)
Sapeornis	Gao et al. (2012); Zheng et al. (2013)
Scansoriopteryx	Czerkas and Feduccia (2014)
Sciurumimus	Rauhut et al. (2012)
Serikornis	Lefèvre et al. (2017)
Shuvuuia	Schweitzer et al. (1999); Saitta et al. (2018)
Similicaudipteryx	He et al. (2008)
Sinocalliopteryx	Ji et al. (2007); Xing et al. (2012); Zheng et al. (2013)
Sinornithosaurus	Xu et al. (1999b, 2001)
Sinosauropteryx	Ji and Ji (1996); Chen et al. (1998); Currie and Chen (2001)
Sordes	Unwin and Bakhurina (1994)
Tianyulong	Zheng et al. (2009)
Tupandactylus	Cincotta et al. (2022)
Velociraptor	Turner et al. (2007)
Wulong	Poust et al. (2020)

Xiaotingia	Xu et al. (2011)
Yanliao anurognathids	Yang et al. (2019)
Yi	Xu et al. (2015)
Yutirannus	Xu et al. (2012)
Zhenyuanlong	Lü and Brusatte (2015)

TABLE S6. References for plumage information in the feathered taxa mentioned in this study. Valuable data was collected as well from Foth and Rauhut (2020).

SUPPLEMENTARY TEXT

Model selection

In this study we used AIC, AICc and LOOCV cross validation for model selection. A rule of thomb commonly used is to treat models within two AIC or AICc units of the best model, as possible viable candidates. We followed such approach in our study. In the case of LOOCV, we considered that models within 10% of the lowest error have a comparable performance to that of the lowest-error model. Following this definition, no less than 17 models can be retained (Table S5). Such model uncertainty, can be incorporated in the results through model averaging, as described in the main text. Model averaging from either AIC/AICc (Fig. S5) or LOOCV mean error (Fig. 8) leads to comparable results.

Taxa added to the phylogenetic tree

To conduct all our analyses, we used a revised version of the phylogenetic tree of Yang et al. (2019). Several new fossil bird and dinosaur taxa with evidence of feather integument have been described over the past few years. These have potential to impact the ancestral state estimation analyses and thus contribute to a better understanding of feather evolution. We incorporated such taxa using the Mesquite software (Maddison and Maddison 2023), on the basis of phylogenetic information from the scientific literature. Additional details are provided below:

- *Sapeornis* and *Jeholornis* were placed according to results from a phylogenetic analysis of Lefèvre et al. (2017). In this analysis, both avialan taxa are closely related, and form a sister taxon to *Rahonavis*, which is more basal.
- The theropod dinosaur *Caihong* was recovered as a sister taxon to *Xiaotingia* in a phylogenetic analysis of Hu et al. (2018).
- *Jianianhualong* was described as a troodontidae (Xu et al. 2017) and as such is placed as a sister taxon of *Jinfengopteryx*, another troondontid dinosaur present in the tree.
- *Wulong* has been recovered as a siter taxon to *Sinornithosaurus* within the Microraptorinae, with *Microraptor* being more basal (Poust et al. 2020).
- IVPP-V-13476 was identified as *Microraptor* (Xu et al. 2003)
- *Dakotaraptor* has been identified as a dromaeosaurinae, sister taxon to the velociraptorinae (DePalma et al. 2015; Currie and Evans 2020).
- *Serikornis* forms a sister taxon to *Eosinopteryx* and *Yi* is closely related to *Epidexipteryx* in a phylogenetic analysis of Lefèvre et al. (2017).
- The phylogenetic placement of DIP-V-15103 is uncertain despite being described as a putative coelurosaur tail (Xing et al. 2016).
- *Apatoraptor* and *Chirostenotes* are closely related, with *Avimimus* being more basal. These form a sister taxon to *Caudipteryx* (Funston and Currie 2016).
- *Jianchangosaurus* was described as a therisinosaur, like *Beipiaosaurus* (Funston and Currie 2016).
- The pterosaur *Tupandactylus* is placed according to the phylogeny of Cincotta et al. (2022).

Supplementary references

Bell P.R., Hendrickx C. 2021. Epidermal complexity in the theropod dinosaur *Juravenator* from the Upper Jurassic of Germany. Palaeontology 64:203–223.

Bell P.R., Hendrickx C., Pittman M., Kaye T.G., Mayr G. 2022. The exquisitely preserved integument of *Psittacosaurus* and the scaly skin of ceratopsian dinosaurs. Commun. Biol. 5:809.

Chen P.-j., Dong Z.-m., Zhen S.-n. 1998. An exceptionally well-preserved theropod dinosaur from the Yixian Formation of China. Nature 391:147–152.

Chiappe L.M., Shu'An J., Qiang J., Norell M.A. 1999. Anatomy and systematics of the Confuciusornithidae (Theropoda: Aves) from the late Mesozoic of northeastern China. Bull. Am. Mus. Nat. Hist. 242.

Christiansen P., Bonde N. 2004. Body plumage in *Archaeopteryx*: a review, and new evidence from the Berlin specimen. C. R. Palevol 3:99–118.

Cincotta A., Nicolaï M., Campos H.B.N., McNamara M., D'Alba L., Shawkey M.D., Kischlat E.-E., Yans J., Carleer R., Escuillié F., Godefroit P. 2022. Pterosaur melanosomes support signalling functions for early feathers. Nature 604:684–688.

Currie P.J., Chen P.-j. 2001. Anatomy of *Sinosauropteryx prima* from Liaoning, northeastern China. Can. J. Earth Sci. 38:1705–1727.

Currie P.J., Evans D.C. 2020. Cranial anatomy of new specimens of *Saurornitholestes langstoni* (Dinosauria, Theropoda, Dromaeosauridae) from the Dinosaur Park Formation (Campanian) of Alberta. Anat. Rec. 303:691–715.

Czerkas S.A., Feduccia A. 2014. Jurassic archosaur is a non-dinosaurian bird. J. Ornithol. 155:841–851.

DePalma R.A., Burnham D.A., Martin L.D., Larson P.L., Bakker R.T. 2015. The first giant raptor (Theropoda: Dromaeosauridae) from the Hell Creek Formation. Paleont. Cont. 14:1–16.

Foth C. 2012. On the identification of feather structures in stem-line representatives of birds: evidence from fossils and actuopalaeontology. Paläontol Z 86:91–102.

Foth C., Rauhut O.W. 2020. The evolution of feathers. Cham: Springer.

Forster C.A., Sampson S.D., Chiappe L.M., Krause D.W. 1998. The theropod ancestry of birds: new evidence from the Late Cretaceous of Madagascar. Science 279:1915–1919.

Funston G.F., Currie P.J. 2016. A new caenagnathid (Dinosauria: Oviraptorosauria) from the Horseshoe Canyon Formation of Alberta, Canada, and a reevaluation of the relationships of Caenagnathidae. J. Vertebr. Paleontol. 36:e1160910.

Funston G.F., Currie P.J. 2021. New material of *Chirostenotes pergracilis* (Theropoda, Oviraptorosauria) from the Campanian Dinosaur Park Formation of Alberta, Canada. Hist. Biol. 33:1671–1685.

Gao C., Chiappe L.M., Zhang F., Pomeroy D.L., Shen C., Chinsamy A., Walsh M.O. 2012. A subadult species of the Early Cretaceous bird *Sapeornis chaoyangensis* and a taxonomic reassessment of sapeornithids. J. Vertebr. Paleontol. 32:1103–1112.

Godefroit P., Cau A., Dong-Yu H., Escuillié F., Wenhao W., Dyke G. 2013. A Jurassic avialan dinosaur from China resolves the early phylogenetic history of birds. Nature 498:359–362.

Godefroit P., Sinitsa S.M., Cincotta A., McNamara M.E., Reshetova S.A., Dhouailly D. 2020. Integumentary Structures in *Kulindadromeus zabaikalicus*, a Basal Neornithischian Dinosaur

from the Jurassic of Siberia. In: Foth C., Rauhut O.W.M., editors. The evolution of feathers. Cham: Springer Nature. p. 47–65.

Han G., Chiappe L.M., Ji S.-A., Habib M., Turner A.H., Chinsamy A.H., Liu X., Han L., 2014. A new raptorial dinosaur with exceptionally long feathering provides insights into dromaeosaurid flight performance. Nat. Commun. 5:4382.

He T., Wang X.-L., Zhou Z.-He 2008. A new genus and species of caudipterid dinosaur from the Lower Cretaceous Jiufotang Formation of Western Liaoning, China. Vert. PalAs. 46:178–189.

Hou L., Zhou Z., Gu Y., Zhang H., 1995. *Confuciusornis sanctus*, a new Late Jurassic sauriurine bird from China. Chin. Sci. Bull. 40:1545–1551.

Hu D., Clarke J.A., Eliason C.M., Qui R., Li Q., Shawkey M.D., Zhao C., D'Alba L., Jiang J., Xu X. 2018. A bony-crested Jurassic dinosaur with evidence of iridescent plumage highlights complexity in early paravian evolution. Nat. Commun. 9:217.

Hu D., Hou L., Zhang L., Xu X. 2009. A pre-*Archaeopteryx* troondontid theropod from China with long feathers on the metatarsus. Nature 461:640–643.

Ji Q., Currie P.J., Norell M.A., Ji S.-A. 1998. Two feathered dinosaurs from northeastern China. Nature 393:753–761.

Ji Q., Ji S.-A. 1996. On the Discovery of the earliest fossil bird in China (*Sinosauropteryx* gen. nov.) and the origin of birds. Chinese Geology 233:30–33.

Ji Q., Ji S.-A. 1997. A Chinese archaeopterygian, *Protarchaeopteryx* gen. nov. Geological Science and Technology 238:38–41.

Ji S.-A., Ji Q., Lu J., Yuan C. 2007. A new giant compsognathid dinosaur with long filamentous integuments from Lower Cretaceous of Northeastern China. Acta Geol. Sin. 81:8–15.

Ji Q., Ji S.A., Lü J.C., You H.L., Chen W., Liu Y.Q., Liu Y.X. 2005. First avialan bird from China. Geol. Bull. China 24:197–210.

Kellner A.W.A., Wang X., Tischlinger H., de Almeida Campos D., Hone D.W.E., Meng X. 2010. The soft tissue of *Jeholopterus* (Pterosauria, Anurognathidae, Batrachognathinae) and the structure of the pterosaur wing membrane. Proc. R. Soc. B 277, 321–329.

Lefèvre U., Cau A., Cincotta A., Hu D., Chinsamy A., Escuillié F., Godefroit P. 2017. A new Jurassic theropod from China documents a transitional step in the macrostructure of feathers. Sci. Nat. 104:74.

Lü J., Brusatte S.L. 2015. A large, short-armed, winged dromaeosaurid (Dinosauria: Theropoda) from the Early Cretaceous of China and its implications for feather evolution. Sci. Rep. 5:11775.

Maddison W. P., Maddison D.R. 2023. Mesquite: a modular system for evolutionary analysis. Version 3.81. http://www.mesquiteproject.org

Mayr G., Peters D.S., Plodowski G., Vogel O. 2002. Bristle-like integumentary structures at the tail of the horned dinosaur *Psittacosaurus*. Naturwiss. 89:361–365.

Mayr G., Pittman M., Saitta E., Kaye T.G., Vinther J. 2016. Structure and homology of *Psittacosaurus* tail bristles. Palaeontology 59:793–802.

O'Connor J., Wang X., Sullivan C., Zheng X., Tubaro P., Zhang X., Zhonghe Z. 2013. Unique caudal plumage of *Jeholornis* and complex tail evolution in early birds. Proc. Natl. Acad. Sci. USA 110:17404–17408.

Poust A.W., Gao C., Varrichio D.J., Wu J., Zhang F. 2020. A new microraptorine theropod from the Jehol Biota and growth in early dromaeosaurids. Anat. Rec. 303:963–987.

Pu H., Kobayashi Y., Lü J, Xu L., Wu Y., Chang H., Zhang J., Jia S. 2013. An unusual basal Therizinosaur dinosaur with an Ornithischian dental arrangement from Northeastern China. PLoS One 8:e63423.

Rauhut O.W.M., Foth C., Tischlinger H., Norell M.A. 2012. Exceptionally preserved juvenile megalosauroid theropod dinosaur with filamentous integument from the Late Jurassic of Germany. Proc. Natl. Acad. Sci. USA 109:11746–11751.

Saitta E.T., Fletcher I., Martin P., Pittman M., Kaye T.G., True L.D., Norell M.A., Abbott G.D., Summons R.E., Penkman K., Vinther J. 2018. Preservation of feather fibers from the Late Cretaceous dinosaur *Shuvuuia deserti* raises concern about immunohistochemical analyses on fossils. Org. Geochem.125:142–151.

Schweitzer M.H., Watt J.A., Avci R., Knapp L., Chiappe L., Norell M., Marshall M. 1999. Beta-keratin specific immunological reactivity in feather-like structures of the Cretaceous Alvarezsaurid, *Shuvuuia deserti*. J. Exp. Zool. B: Mol. Dev. Evol. 285:146–157.

Turner A.H., Makovicky P.J., Norell M.A 2007. Feather quill knobs in the dinosaur *Velociraptor*. Science 317:1721.

Unwin D.M., Bakhurina N.N. 1994. *Sordes pilosus* and the nature of the pterosaur flight apparatus. Nature 371:62–64.

van der Reest A.J., Wolfe A.P., Currie P.J. 2016. A densely feathered ornithomimid (Dinosauria: Theropoda) from the Upper Cretaceous Dinosaur Park Formation, Alberta, Canada. Cret. Res. 58:108–117.

Wang M., O'Connor J.K., Xu X., Zhou Z. 2019. A new Jurassic scansoriopterygid and the loss of membranous wings in theropod dinosaurs. Nature 259:256–259.

Wang X., Zhou Z., Zhang F., Xu X. 2002. A nearly completely articulated rhamphorhynchoid pterosaur with exceptionally well-preserved wing membranes and "hairs" from Inner Mongolia, northeast China. Chin. Sci. Bull. 47:226–230.

Xing L., Bell P.R., Persons IV W.S., Ji S., Miyashita T., Burns M.E., Ji Q., Currie P.J. 2012. Abdominal contents from two large Early Cretaceous Compsognathids (Dinosauria: Theropoda) demonstrate feeding on Confuciusornithids and Dromaeosaurids. PLoS One 7:e44012.

Xing L., McKellar R.C., Xu X., Li G., Bai M., Persons IV W.S., Miyashita T., Benton M.J., Zhang J., Wolfe A.P., Yi Q., Tseng K., Ran H., Currie P.J. 2016. A feathered dinosaur tail with primitive plumage trapped in mid-Cretaceous amber. Curr. Biol. 26:3352–3360.

Xu X., Currie P., Pittman M., Xing L., Meng Q., Lü J., Hu D., Yu C. 2017. Mosaic evolution in an asymmetrically feathered troodontid dinosaur with transitional features. Nat. Commun. 8:14972.

Xu X., Norell M.A., Kuang X., Wang X., Zhao Q., Jia C. 2004. Basal tyrannosauroids from China and evidence for protofeathers in tyrannosauroids. Nature 431:680–684.

Xu X., Tang Z.-l., Wang X.-l. 1999a. A therizinosauroid dinosaur with integumentary structures from China. Nature 399:350–354.

Xu X., Wang X.-L., Wu X.-C. 1999b. A dromaeosaurid dinosaur with a filamentous integument from the Yixian Formation of China. Nature 401:262–266.

Xu X., Wang K., Zhang K., Ma Q., Xing L., Sullivan C., Hu D., Cheng S., Wang S. 2012. A gigantic feathered dinosaur from the Lower Cretaceous of China. Nature 484:92–95.
Xu X., You H., Du K., Han F. 2011. An *Archaeopteryx*-like theropod from China and the origin of Avialae. Nature 475:465–470.
Xu X., Zhang F. 2005. A new maniraptoran dinosaur from China with long feathers on the metatarsus. Naturwiss. 92:173–177.
Xu X., Zheng X., Sullivan C., Wang X., Xing L., Wang Y., Zhang X., O'Connor J.K., Zhang F., Pan Y. 2015. A bizarre Jurassic maniraptoran theropod with preserved evidence of membranous wings. Nature 521:70–73.
Xu X., Zheng X., You H. 2009. A new feather type in a nonavian theropod and the early evolution of feathers. Proc. Natl. Acad. Sci. U.S.A. 106:832–834.
Xu X., Zheng X., You H. 2010. Exceptional dinosaur fossils show ontogenetic development of early feathers. Nature 464:1338–1341.
Xu X., Zhou Z.-h., Prum R.O. 2001. Branched integumental structures in *Sinornithosaurus* and the origin of feathers. Nature 410: 200–204.
Xu X., Zhou Z., Wang X., Kuang X., Zhang F., Du X. 2003. Four-winged dinosaurs from China. Nature 421:335–340.
Yang, Y., Jiang B., McNamara M.E., Kearns S.L., Pittman M., Kaye T.G., Orr P.J., Xu X., Benton M.J. 2019. Pterosaur integumentary structures with complex feather-like branching. Nat. Ecol. Evol. 3:24–30.
Zelenitsky D.K., Therrien F., Erickson G.M., DeBuhr C.L., Kobayashi Y., Eberth D.A., Hadfield F. 2012. Feathered non-avian dinosaurs from North America provide insight into wing origins. Science 338:510–514.
Zhang F., Zhou Z., Xu X., Wang X., Sullivan C. 2008. A bizarre Jurassic maniraptoran from China with elongate ribbon-like feathers. Nature 455:1105–1108.
Zheng X.-T., You H.-L., Xu X., Dong Z.-M. 2009. An Early Cretaceous heterodontosaurid dinosaur with filamentous integumentary structures. Nature 458:333–336.
Zheng X., Zhou Z., Wang X., Zhang F., Zhang X., Wang Y., Wei G., Wang S., Xu X. 2013. Hind wings in basal birds and the evolution of leg feathers. Science 339:1309–1312.
Zhou Z., Hou L. 1998. *Confuciusornis* and the early evolution of birds. Vert. PalAs. 36:136–146.
Zhou Z.-H., Wang X.-L. 2000. A new species of *Caudipteryx* from the Yixian Formation of Liaoning, northeast China. Vert. PalAs. 38:111–127.
